# Floristic survey of vascular plants of a poorly known area in the Brazilian Atlantic Forest (Flona do Rio Preto, Espírito Santo)

**DOI:** 10.3897/BDJ.10.e75910

**Published:** 2022-01-19

**Authors:** Anderson Alves-Araújo, Marina M Moreira, Tatiana T Carrijo, Lúcia G Lohmann, Adriana Q Lobão, Alana F Scheidegger, Aline D Firmino, Aline Vieira de Melo Silva, Álvaro Nepomuceno, Amélia C Tuler, André MA Amorim, André LC Moreira, Braz AP Cosenza, Brenno G Sossai, Christian Silva, Claudia R Lopes, Daniele Monteiro, Dayvid R Couto, Duane F Lima, Eduardo C Dalcin, Eliana Ramos, Elton J Lírio, Fatima Salimena, Felipe Alves de Oliveira, Fernanda RM Fraga, Filipe Torres-Leite, Guilherme M Antar, Gustavo H Shimizu, Haroldo C Lima, Herison Medeiros, Jaquelini Luber, Jheniffer A Christ, João Lanna, João Paulo F Zorzanelli, Joelcio Freitas, José FB Pastore, José IM Melo, Juliana Paula-Souza, Juliana RPM Oliveira, Leandro C Pederneiras, Leandro Freitas, Leandro L Giacomin, Leonardo D Meireles, Luis AE Silva, Luiz JS Pinto, Luiz Menini Neto, Marcelo Trovó, Mário L Garbin, Marli P Morim, Michel Ribeiro, Nelson TL Pena, Paulo H Labiak, Pedro H Cardoso, Pedro L Viana, Pedro LR Moraes, Quélita S Moraes, Raquel F Zorzanelli, Renara N Amaral, Renata C Asprino, Renato Goldenberg, Ricardo Magnago, Ricardo S Couto, Sandrine C Dutra, Saúl E Hoyos-Gómez, Tamara AF Vieira, Thiago B Flores, Valquíria F Dutra, Víctor S Miranda, Vitor C Manhães, Rafaela C Forzza

**Affiliations:** 1 Universidade Federal do Espírito Santo, São Mateus, Brazil Universidade Federal do Espírito Santo São Mateus Brazil; 2 Universidade Federal do Espírito Santo, Vitória, Brazil Universidade Federal do Espírito Santo Vitória Brazil; 3 Universidade Federal do Espírito Santo, Alegre, Brazil Universidade Federal do Espírito Santo Alegre Brazil; 4 Universidade de São Paulo, São Paulo, Brazil Universidade de São Paulo São Paulo Brazil; 5 Universidade Federal Fluminense, Rio de Janeiro, Brazil Universidade Federal Fluminense Rio de Janeiro Brazil; 6 Universidade Federal de Pernambuco, Recife, Brazil Universidade Federal de Pernambuco Recife Brazil; 7 Instituto Nacional da Mata Atlântica, Santa Teresa, Brazil Instituto Nacional da Mata Atlântica Santa Teresa Brazil; 8 Universidade Estadual de Santa Cruz, Ilhéus, Brazil Universidade Estadual de Santa Cruz Ilhéus Brazil; 9 Universidade de Brasília, Distrito Federal, Brazil Universidade de Brasília Distrito Federal Brazil; 10 Universidade do Estado de Minas Gerais, Belo Horizonte, Brazil Universidade do Estado de Minas Gerais Belo Horizonte Brazil; 11 Universidade do Estado de Santa Catarina, Laguna, Brazil Universidade do Estado de Santa Catarina Laguna Brazil; 12 Jardim Botânico do Rio de Janeiro, Rio de Janeiro, Brazil Jardim Botânico do Rio de Janeiro Rio de Janeiro Brazil; 13 Universidade Estadual do Norte Fluminense, Campos dos Goytacazes, Brazil Universidade Estadual do Norte Fluminense Campos dos Goytacazes Brazil; 14 Universidade Federal de Santa Catarina, Florianópolis, Brazil Universidade Federal de Santa Catarina Florianópolis Brazil; 15 Universidade Federal de Juiz de Fora, Juiz de Fora, Brazil Universidade Federal de Juiz de Fora Juiz de Fora Brazil; 16 Universidade Estadual de Campinas, Campinas, Brazil Universidade Estadual de Campinas Campinas Brazil; 17 Universidade Federal do Rio de Janeiro, Rio de Janeiro, Brazil Universidade Federal do Rio de Janeiro Rio de Janeiro Brazil; 18 Universidade Estadual de Feira de Santana, Feira de Santana, Brazil Universidade Estadual de Feira de Santana Feira de Santana Brazil; 19 Universidade Estadual da Paraíba, Campina Grande, Brazil Universidade Estadual da Paraíba Campina Grande Brazil; 20 Universidade Federal do Oeste do Pará, Santarém, Brazil Universidade Federal do Oeste do Pará Santarém Brazil; 21 Universidade Federal de Viçosa, Viçosa, Brazil Universidade Federal de Viçosa Viçosa Brazil; 22 Universidade Federal do Paraná, Curitiba, Brazil Universidade Federal do Paraná Curitiba Brazil; 23 Museu Paraense Emílio Goeldi, Belém, Brazil Museu Paraense Emílio Goeldi Belém Brazil; 24 Universidade Estadual Paulista Júlio de Mesquita Filho, Rio Claro, Brazil Universidade Estadual Paulista Júlio de Mesquita Filho Rio Claro Brazil; 25 Universidad de Antioquia, Medelín, Colombia Universidad de Antioquia Medelín Colombia

**Keywords:** conservation, endemism, new records, threatened species

## Abstract

**Background:**

The Atlantic Forest is one of the most threatened biomes in the world. Despite that, this biome still includes many areas that are poorly known floristically, including several protected areas, such as the "Floresta Nacional do Rio Preto" ("Flona do Rio Preto"), located in the Brazilian State of Espírito Santo. This study used a published vascular plant species list for this protected area from the "Catálogo de Plantas das Unidades de Conservação do Brasil" as the basis to synthesise the species richness, endemism, conservation and new species occurrences found in the "Flona do Rio Preto".

**New information:**

The published list of vascular plants was based on field expeditions conducted between 2018 and 2020 and data obtained from herbarium collections available in online databases. Overall, 722 species were documented for the "Flona do Rio Preto", 711 of which are native to Brazil and 349 are endemic to the Atlantic Forest. In addition, 60 species are geographically disjunct between the Atlantic and the Amazon Forests. Most of the documented species are woody and more than 50% of these are trees. Twenty-three species are threatened (CR, EN and VU), while five are Data Deficient (DD). Thirty-two species are new records for the State of Espírito Santo. Our results expand the knowledge of the flora of the Atlantic Forest and provide support for the development of new conservation policies for this protected area.

## Introduction

The Atlantic Forest houses one of the highest levels of species diversity and endemism in Brazil (Werneck et al. 2011). Despite that, the Atlantic Forest has been reduced to small and disconnected forest remnants, with only ca. 28% of its original forest cover remaining, of which less than half are protected (Rezende et al. 2018).

Unique in its occupation history, the State of Espírito Santo is completely inserted within the Atlantic Forest and exhibits, from the coast to the mountains, contrasting vegetation types (Dutra et al. 2015). Garbin et al. (2017) described the vision of many naturalists who passed through Espírito Santo in the 19^th^ and early 20^th^ centuries, providing a nice historical overview for the State. These naturalists highlighted the exuberance and distinction between the vegetation located in the southern and northern portions of the Doce River.

Six vegetation types are recognised in Espírito Santo: (i) dense and open ombrophilous forests, (ii) seasonal semi-deciduous forests, (iii) savannahs, (iv) "restingas", (v) mangroves and (vi) ecological refuges (Garbin et al. 2017). These vegetation types are distributed through two main geological zones (IPEMA 2005): A central-southern zone comprising deep montane valleys and a central-northern zone with "tabuleiro" forests. This vegetation mosaic is part of the "Corredor Central da Mata Atlântica" (Aguiar and Marques 2001), one of the regions with highest plant endemism in Brazil (Prado et al. 2003, Ayres et al. 2005, IPEMA 2005).

Floristic inventories were produced for the State of Espírito Santo during the last 30 years (e.g. Peixoto and Gentry 1990, Peixoto et al. 2008, Thomaz 2010, Rolim et al. 2016). The most comprehensive checklist of the angiosperms for the Espírito Santo was produced, based on a synthesis of online data (Dutra et al. 2015). These efforts resulted in the publication of several taxonomic and floristic studies for selected plant families in subsequent years (e.g. Luber et al. 2016, Pena and Alves-Araújo 2017, Silva et al. 2017, Chagas et al. 2017, Souza et al. 2017, Araújo et al. 2018, Torres-Leite et al. 2018, Moreira et al. 2020a, Moreira et al. 2020b). Despite that, the State includes multiple knowledge gaps and botanical data remain scattered (Garbin et al. 2017). A full understanding of the forest dynamics of Espírito Santo depends on more detailed floristic inventories and vegetation data for the State. Information of this nature is crucial to fill knowledge gaps and establish sound conservation policies.

The "Catálogo de Plantas das Unidades de Conservação do Brasil" (https://catalogo-ucs-brasil.jbrj.gov.br/) was launched online in 2018 to contribute to the knowledge of the Brazilian protected areas. This digital platform provides comprehensive lists of land plants from the Brazilian protected areas, providing information about species correct names, conservation status, native/non-native status and digital images. The catalogue currently contains plant lists from 18 conservation units located in different Brazilian phytogeographic domains (e.g. Caatinga, Cerrado, Atlantic Forest and Amazon). The checklist of the vascular plants of the "Parque Nacional do Itatiaia" (Carrijo et al. 2018, Moreira et al. 2020a) and "Parque Nacional do Caparaó" (Moreira et al. 2020b, Carrijo et al. 2021) were the first to be launched, while the checklist of the vascular plants of the "Flona do Rio Preto" was recently added (Carrijo et al. 2021).

The "Flona do Rio Preto" was officially created in 1990 and is located in the northern portion of Espírito Santo (IBGE 2012). This protected area is mainly covered by dense ombrophilous lowland forest (IBGE 2012), despite the vegetation from the northern Espírito Santo having been classified as seasonal evergreen forest (Rolim et al. 2016). Historically, the area has undergone loss of natural vegetation due to anthropogenic disturbances, such as logging, grazing and fires, followed by the cultivation of *Eucalyptus* and sugar cane (Souza and Resende 1999). Despite these threats, the "Flona do Rio Preto" still offers good quality environments and floristic integrity. However, information about the local flora is restricted to the first forest inventory which focused on the documentation of woody trees and climbers (Souza and Resende 1999). Therefore, the vascular plant richness and composition of this protected area is still underestimated, especially due to a lack of documentation of herbaceous and shrubby species. Here, we summarise the information from a recent inventory (Carrijo et al. 2021) and present information about species richness, endemism, conservation and new species records from the "Flona do Rio Preto."

## Sampling methods

### Study extent

**Figure 1.**
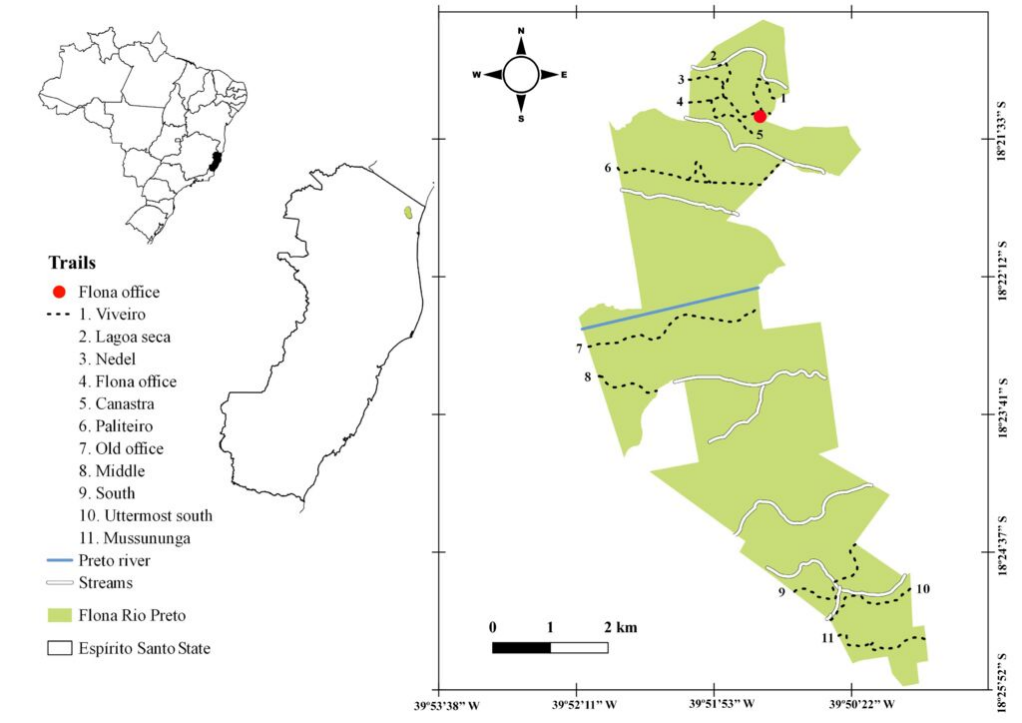
Map showing the location of the "Flona do Rio Preto," Espírito Santo, Brazil.

During this project, we undertook field expeditions to the "Flona do Rio Preto" fortnightly or monthly, from March 2018 to February 2020. Sampling covered all physiognomies (Fig. 1) and was conducted by walking randomly across trails (Fig. 2). Collected materials were dried following standard methods for plant taxonomy (Peixoto and Maia 2013) and deposited at the Herbarium VIES (acronym from Thiers 2021), where samples were also digitised.

**Figure 3.**
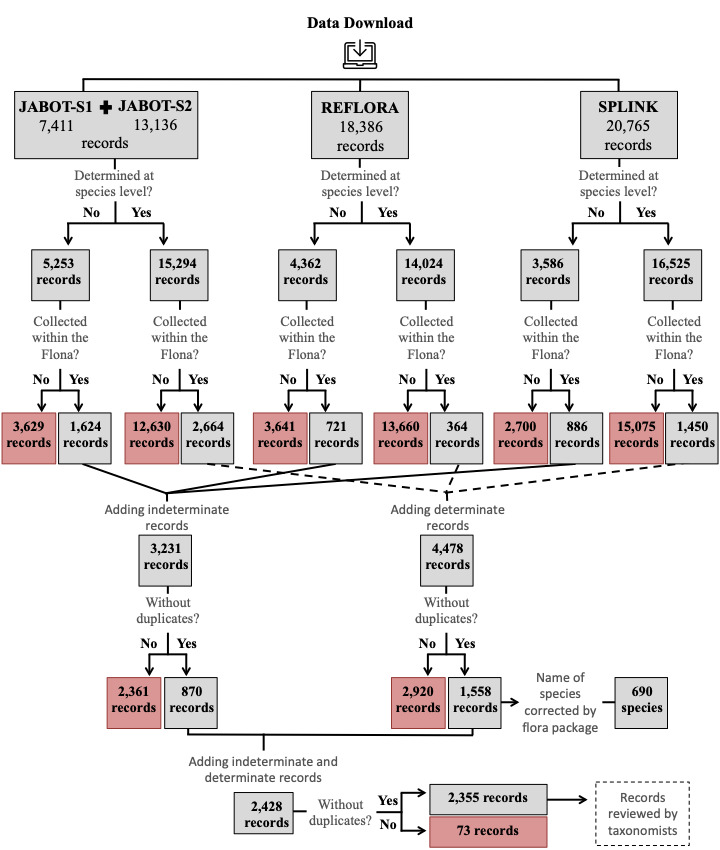
Stages of data cleaning performed in R software to obtain a list of vascular plant species of the "Flona do Rio Preto," Brazil. Specimens kept on the list are shown in grey; specimens removed from the list are shown in red.

In addition to the data collected in the field, we also downloaded information from three online databases: JABOT ("Jardim Botânico do Rio de Janeiro," JBRJ, http://www.jbrj.gov.br/jabot), REFLORA ("Herbário Virtual Reflora," http://reflora.jbrj.gov.br) and Splink ("INCT Herbário Virtual da Flora e dos Fungos," http://inct.splink.org.br) (Fig. 1) We performed searches in each database on 1 October 2020, using the following filters: **Search 1**: Locality = "Rio Preto" (JABOT = 7,411 specimens; REFLORA = 18,386; Splink = 20,765); and **Search 2**: Locality = "Conceição da Barra" (JABOT = 13,136 specimens). Together, these searches led to a total of 59,698 specimens (Fig. 3).

To obtain a list of species with currently accepted nomenclature, we manually selected all specimens identified to the species level, which led to the following: **Search 1**: JABOT determined = 5,237, undetermined = 2,174; REFLORA determined = 14,024, undetermined = 4,362; and Splink determined = 16,525, undetermined = 3,586; **Search 2**: JABOT determined = 10,057, undetermined = 3,079 (Fig. 3). We then selected specimens for which the localities fall within the area covered by the "Flona do Rio Preto" and removed duplicates, based on the catalogue code, collector name and number and the year when the sample was collected (Fig. 3). For the list of determined specimens, we corrected and updated species names using the function *get.taxa* from the package flora (Carvalho 2017) in R 4.0.2 (R Development Core Team 2019). This function compared the names in our list with those from the Flora do Brasil 2020 (http://floradobrasil.jbrj.gov.br). After these corrections, we had taxonomists check a preliminary list with 690 determined species (1,558 specimens) and 870 undetermined specimens using images available from online databases (Fig. 3). When a taxonomist modified a plant species name, at least one specimen of that species was updated in the REFLORA database, but not in Splink. Infraspecific taxonomic categories or hybrids were not considered. The final list was published by Carrijo et al. (2021) and is available at (https://catalogo-ucs-brasil.jbrj.gov.br/).

**Life forms**

We obtained information on life forms from the Flora do Brasil 2020 (http://floradobrasil.jbrj.gov.br) through the function get_lifeform of the flora package (Carvalho 2017). Considering that the Flora do Brasil 2020 indicated more than one life form for several species, we conducted searches using three categories: (i) woody species (trees, shrubs and subshrubs, excluding climbers); (ii) herbaceous species (including palms and excluding climbers); and (iii) climbers (woody and/or herbaceous). When species were simultaneously assigned as a "climber" and "shrub" in the database, we chose to classify them as a "shrub" since "scandent shrubs" are often mistakenly treated as "climbers." As far as substrates are concerned, plants were classified as aquatic, epiphytic, hemi-epiphytic, hemiparasitic, mycoheterotrophic or terrestrial.

**Endemic and threatened species**

We classified all species as native or non-native and endemic or non-endemic to Brazil using information from the Flora do Brasil 2020 (http://floradobrasil.jbrj.gov.br). Threat categories were assigned according to the Brazilian National Red List (CNCFlora; http://www.cncflora.jbrj.gov.br/portal). For binomials not included in the Flora do Brasil 2020, their native/non-native or endemic/non-endemic status was obtained from taxonomists and experts in those groups. We considered as non-native all species indicated as cultivated or naturalised within the Flora do Brasil 2020 (http://floradobrasil.jbrj.gov.br). We classified species as endemic to the Atlantic Forest when their distribution was classified as restricted to this phytogeographic domain in the Flora do Brasil 2020 (http://floradobrasil.jbrj.gov.br). We obtained this information using the function get_domains of the flora package (Carvalho 2017).

## Geographic coverage

### Description

The "Floresta Nacional do Rio Preto" ("Flona do Rio Preto") is located in the Municipality of Conceição da Barra, in the northern portion of the Brazilian State of Espírito Santo (ES), in south-eastern Brazil (Souza and Resende 1999) (Fig. 2). This protected area encompasses approximately 2,830 ha and includes different vegetation types (see Garbin et al. 2017), namely: (i) seasonally flooded open field, (ii) native fields (in Portuguese, "Campo nativo"), (iii) non-floodable forests, (iv) seasonally flooded forests, (v) "Mussununga" and (vi) seasonally flooded swampy areas (Fig. 1). The climate is tropical Aw (rainy tropical; Álvares et al. 2013), with total annual rainfall between 1,210 mm and 1,259 mm and an average annual temperature of 22°C or higher (Souza and Resende 1999).

### Coordinates

-18.445 and -18.342 Latitude; -39.874 and -39.817 Longitude.

## Taxonomic coverage

### Description

The list of vascular plants of the "Flona do Rio Preto" contains a total of 722 species (683 angiosperms, 38 ferns and one lycophyte) belonging to 425 genera (398 angiosperms, 26 ferns and one lycophyte) and 126 families (111 angiosperms, 14 ferns and one lycophyte). We found no records for gymnosperms at the study site during fieldwork nor in the online databases.

The vascular plant list of the "Flona do Rio Preto" exhibits levels of taxonomic richness that are similar to those documented in other floristic inventories conducted in the northern portions of Espírito Santo. For example, a study conducted in an area of alluvial floodplain of the Doce River found 408 angiosperm species (Rolim et al. 2016), while the plant list of the "Reserva Natural da Vale" listed 2,095 vascular plant species (1,999 angiosperms, 93 ferns and three lycophytes) (Rolim et al. 2016, Sylvestre et al. 2016). The "Reserva Natural da Vale" encompasses an area of 23,000 ha, representing one of the largest conservation units within the Atlantic Forest (Rolim et al. 2016).

**Figure 4.**
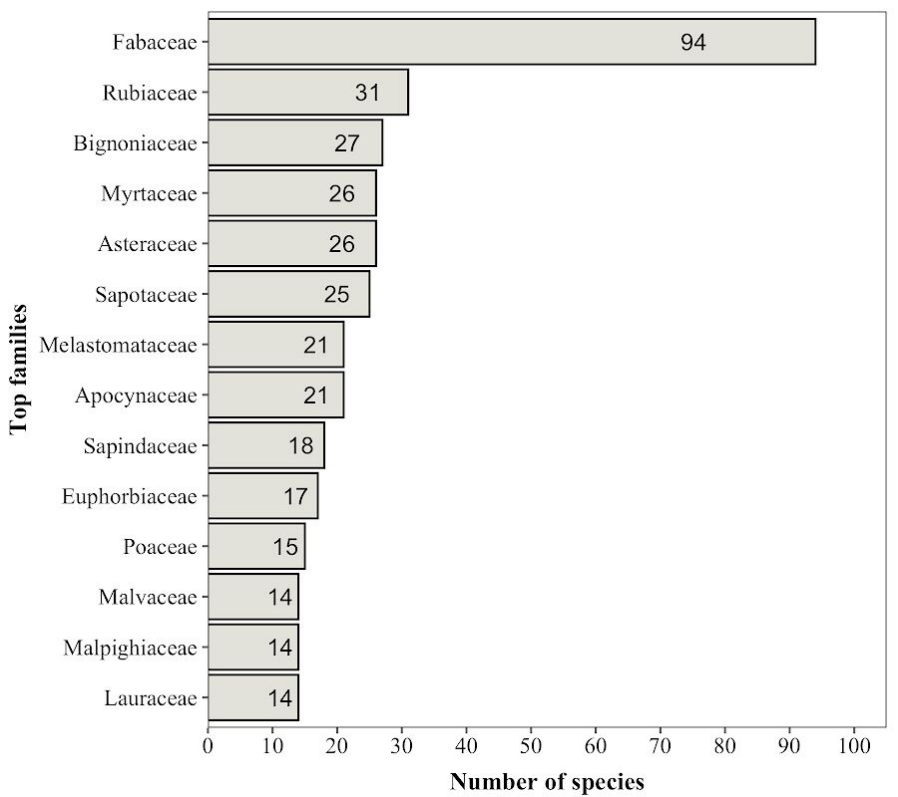
Richest families of major groups of plants recorded in the "Flona do Rio Preto," Brazil. Values inside bars indicate species numbers.

Overall, the "Flona do Rio Preto" houses approximately 11% of the vascular plants that occur in Espírito Santo (Flora do Brasil 2020 2020). The richest plant families in the "Flona do Rio Preto" are Fabaceae, Rubiaceae, Bignoniaceae, Myrtaceae, Asteraceae, Sapotaceae, Melastomataceae, Apocynaceae, Sapindaceae, Euphorbiaceae, Poaceae, Lauraceae, Malpighiaceae and Malvaceae, respectively (Fig. 4). These families comprise 50% (i.e. 363 spp.) of all species found in the area. Forty-two families are represented by a single species. These findings are consistent with floristic inventories conducted in other forests remnants located in the Brazilian States of Espírito Santo and Bahia (Paula 2006, Amorim et al. 2008, Siqueira et al. 2014, Rolim et al. 2016), where the same richest families are generally documented. Despite the high floristic similarity documented in those inventories, only four species of Orchidaceae were found in the "Flona do Rio Preto," contrasting with the 103 spp. of Orchidaceae documented by Rolim et al. (2016). The low number of orchid species documented might be due to a shorter sampling period, incipient sampling efforts for epiphytes, in general, at the "Flona do Rio Preto," differences in climatic conditions or even the historical conservation of the "Flona do Rio Preto."

## Traits coverage

**Life forms**

In total, 447 species (61.9%) occurring in the "Flona do Rio Preto" are woody (trees, shrubs and subshrubs, excluding climbers), of which 219 are trees. Herbs (excluding climbing plants) are represented by 164 species (22.7%) and climbers (herbaceous or woody) are composed of 111 species (15.4%). The richest woody families are Fabaceae (82 spp.), Myrtaceae and Rubiaceae (26 spp. each), Sapotaceae (25 spp.), Melastomataceae (18 spp.) and Lauraceae (14 spp.). The richest families of herbs are Poaceae (15 spp.), Marantaceae (13 spp.), Arecaceae (9 spp.), Asteraceae (8 spp.) and Cyperaceae (7 spp.). The most diverse liana families are Bignoniaceae (19 spp.), Fabaceae (12 spp.), Apocynaceae (11 spp.), Passifloraceae (10 spp.), Malpighiaceae and Sapindaceae (9 spp. each). In total, 682 species (94.5%) are terrestrial, followed by aquatic, epiphytes, hemi-epiphytes, mycoheterotrophic plants and hemiparasites which together comprise 40 species (5.5%). Bromeliaceae (8 spp.) and Cyperaceae (3 spp.) emerged as the richest families amongst epiphytes and aquatic plants, respectively.

**Endemic and threatened species**

The list of the "Flona do Rio Preto" includes 711 native and 11 non-native species to Brazil. Some non-native species were found in the "Flona do Rio Preto," but were not included in the final list because no herbarium records were found, such as *Mangiferaindica* L. (Anacardiaceae); *Cocosnucifera* L. (Arecaceae); *Terminalia catappa* L. (Combretaceae); *Acaciamangium* Willd. and *Acaciaauriculiformis* A.Cunn. ex Benth. (Fabaceae); *Artocarpusheterophyllus* Lam. (Moraceae); *Eucalyptus* L’Hér. spp., *Psidiumguajava* L., *Syzygiumcumini* (L.) Skeels and *Syzygiummalaccense* (L.) Merr. & L.M.Perry (Myrtaceae). Despite that, we highlight the occurrence of these taxa in the "Flora do Rio Preto" as the occurrence of these species represents important information for the establishment of sound management policies (Moro et al. 2012). Amongst the species recorded at the "Flona do Rio Preto," 349 are endemic (Figs 5, 6, 7) and 373 are non-endemic to Brazil. Furthermore, 253 species are endemic to the Brazilian Atlantic Forest, while 464 are non-endemic to this biome.

Thirty-five percent of the species that occur in the "Flona do Rio Preto" are endemic to the Atlantic Forest. Another 60 species that occur in the studied area (8.3%) have a known geographical distribution that is disjunct between the Atlantic and Amazon Forests. Amongst the plant families with the highest number of Amazon-Atlantic Forest disjunct species are the Fabaceae (6 spp.), Pteridaceae (6 spp.), Sapindaceae (4 spp.), Euphorbiaceae (3 spp.) and Moraceae (3 spp.). In addition, 46 of the species recorded in the "Flona do Rio Preto" are disjunctly distributed between the Atlantic Forest and the Cerrado, while 17 species are disjunctly distributed between the Atlantic Forest and Caatinga. The remaining 346 species (48%) are widely distributed throughout Brazil.

Overall, the final list of vascular plants includes 55 species not cited in the Flora do Brasil 2020 for Espírito Santo (Suppl. material 1). Amongst these, 23 were listed in previous floristic studies carried out in Espírito Santo (Dutra et al. 2015, Souza et al. 2016) (Suppl. material 1). Therefore, 32 species, belonging to 29 genera and 22 families, represent new records for the State (Suppl. material 1).

Based on the Brazilian National Red List, the "Flona do Rio Preto" includes 76 species considered as Least Concern (LC), 11 as Endangered (EN), 11 as Vulnerable (VU), 12 as Near Threatened (NT), one as Critically Endangered (CR) and five as Data Deficient (DD). In other words, the "Flona do Rio Preto" houses 23 threatened species (CR, EN and VU) and five Data Deficient species (Table 1, Figs 5c, 6b, 7b, f). These species belong to 15 families, with Sapotaceae including the highest number of threatened species (7 spp.) followed by Chrysobalanaceae, Fabaceae (3 spp.), Rubiaceae (3 spp.) and Bignoniaceae (2 spp.); ten other families included one threatened species (Table 1). In total, 606 species (84%) that occur in the "Flona do Rio Preto" have not been evaluated by the Brazilian National Red List.

## Temporal coverage

### Notes

**Temporal and spatial coverage**

The taxonomists, associated with this project, verified a total of 1,959 specimens. These specimens are deposited in nine Brazilian Herbaria (BHCB, CEN, CESJ, ESA, HUEMG, RB, SAMES, VIC and VIES) with VIES hosting 80% (1,572) of these specimens (acronyms follow Thiers 2021). The examined specimens were collected by thirty-four collectors, with R. Nichio-Amaral having collected the highest number of specimens (229 specimens), followed by A. Nepomuceno (179), B.N. Mello (175), A. Luiza (164), S.C. Dutra (153), B.G. Sossai (141), "Grupo de Coletores do Núcleo Juçara" (128), R.M. Oliveira (113), B.S. Mendes (93), V.S. Miranda (93) and A.F.A. Scheidegger (86).

Field sampling in the "Flona do Rio Preto" started in 1953, even before its creation and extended until 2020. The years of 2019 (1,015 specimens), 2018 (257 specimens), 2020 (184 specimens) and 1995 (156 specimens) were the most intensively sampled. Collections conducted between 2018 and 2020 highlight the importance of the project "Rediscovering species threatened with extinction: Basis for management and information access," for increased knowledge about the flora of the "Flona do Rio Preto."

## Usage licence

### Usage licence

Creative Commons Public Domain Waiver (CC-Zero)

## Data resources

### Data package title

Floristic survey of vascular plants of a poorly known area in the Brazilian Atlantic Forest (Flona do Rio Preto, Espírito Santo)

### Resource link

https://doi.org/10.5281/zenodo.5824423

### Number of data sets

1

### Data set 1.

#### Data set name

lista-flona-rio-preto.tsv

#### Data format

tsv

#### Number of columns

13

#### Download URL

https://zenodo.org/record/5824423/files/lista-flona-rio-preto.tsv?download=1

**Data set 1.** 

Column label	Column description
fieldNotes	Name of the National Conservation Unit
Grupos	Taxonomic group
family	Botanical families names
genus	Botanical genus names
specificEpithet	Botanical species epithet names
scientificNameAuthorship	Species authors' names
scientificName (according to Flora do Brasil 2020)	Táxon according to Flora do Brasil 2020 project
barcode	Barcode of the specimen
Banco de Origem	Database of origin
otherCatalogNumbers	Catalogue number of the specimen
collectionCode	Acronym of the collection
recordedBy	Name of the specimen collector
recordNumber	Collector number

## Additional information

**Conclusions and prospects**

The list of the vascular plants of the "Flona do Rio Preto" expands the knowledge of the flora of the Atlantic Forest and provides important data for the development of sound conservation policies. Our study documented 253 endemic Atlantic Forest taxa at the "Flona do Rio Preto" and 32 new records of angiosperms for the State of Espírito Santo. The small number of epiphytes found in the studied area suggests that the "Flona do Rio Preto" has a vegetation type that does not match an Ombrophilous Dense Forest, the phytophysiognomy under which it was previously classified. The species list, provided here, contributes important information for an improved vegetation classification.

## Supplementary Material

D5D80F9C-745C-5EC8-B232-C606C4BDD59E 10.3897/BDJ.10.e75910.suppl1 Supplementary material 1 New records for the State of Espírito Santo Data type taxonomic Brief description List of new species records for the State of Espírito Santo (ES), according to Flora do Brasil 2020. File: oo_593551.tsv https://binary.pensoft.net/file/593551 Alves-Araújo et al. (2021)

## Figures and Tables

**Figure 2a.**
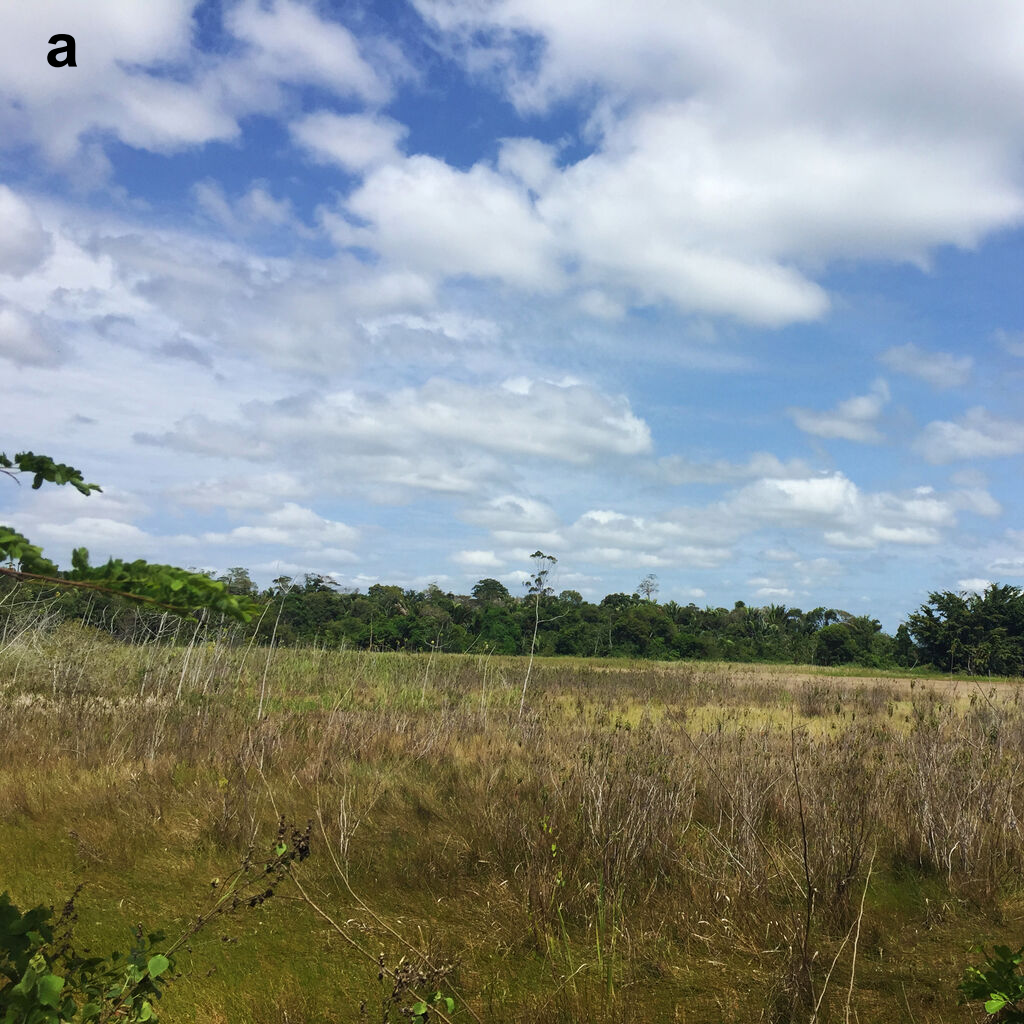
Seasonally flooded open field

**Figure 2b.**
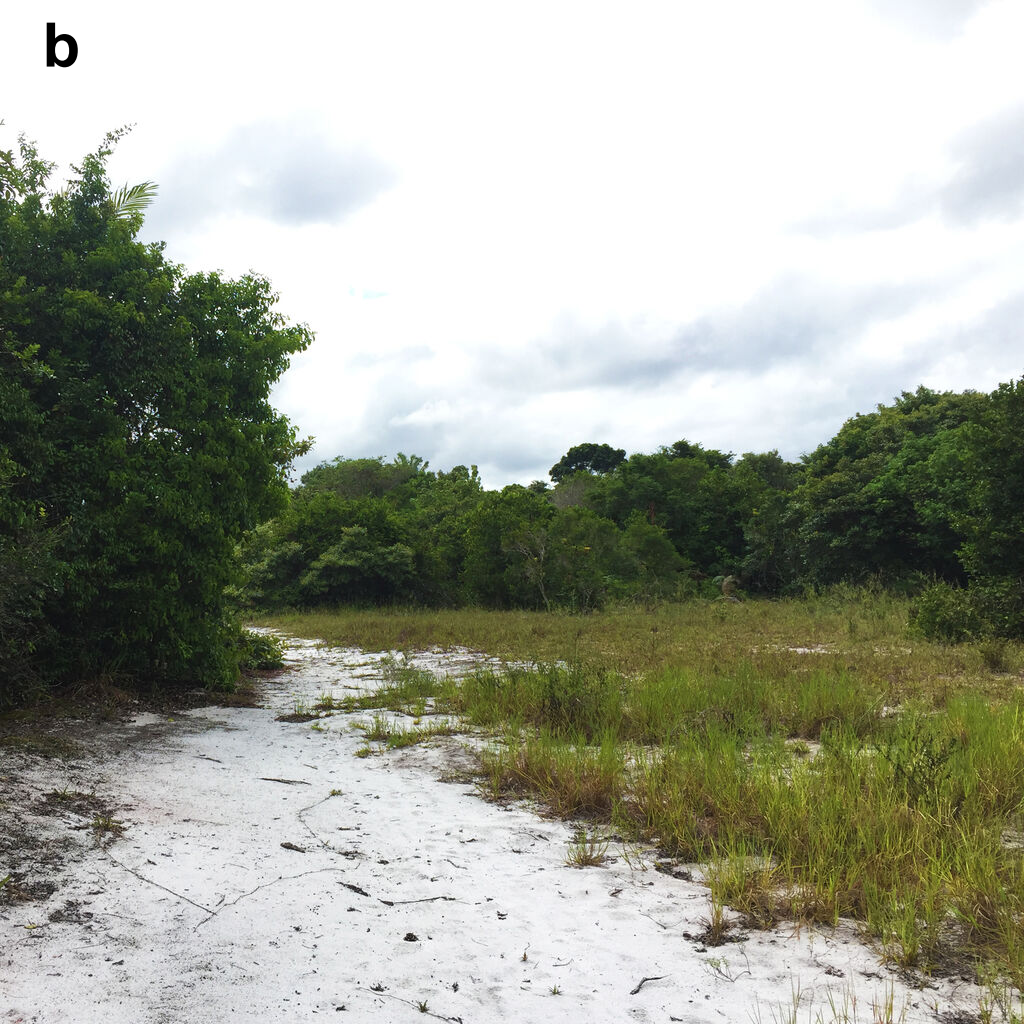
Campos nativos’

**Figure 2c.**
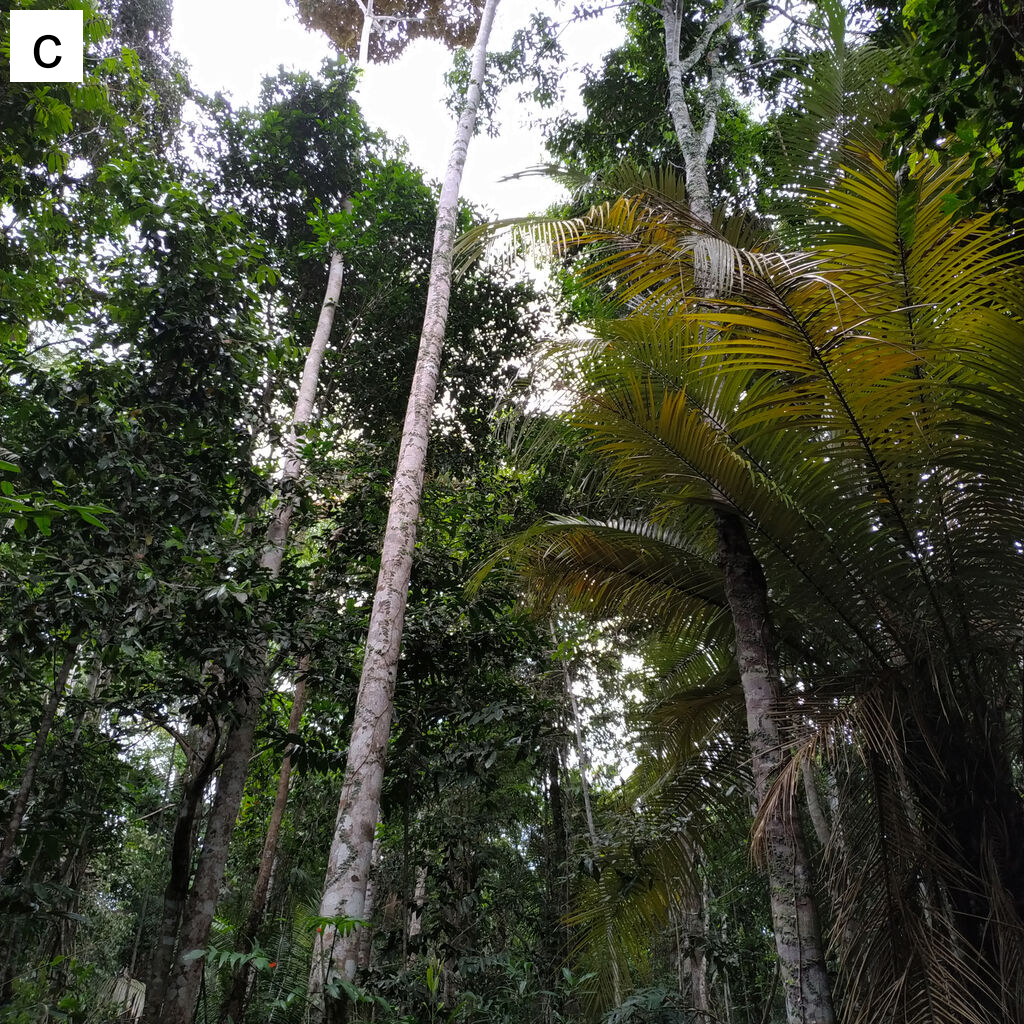
Non-floodable forest

**Figure 2d.**
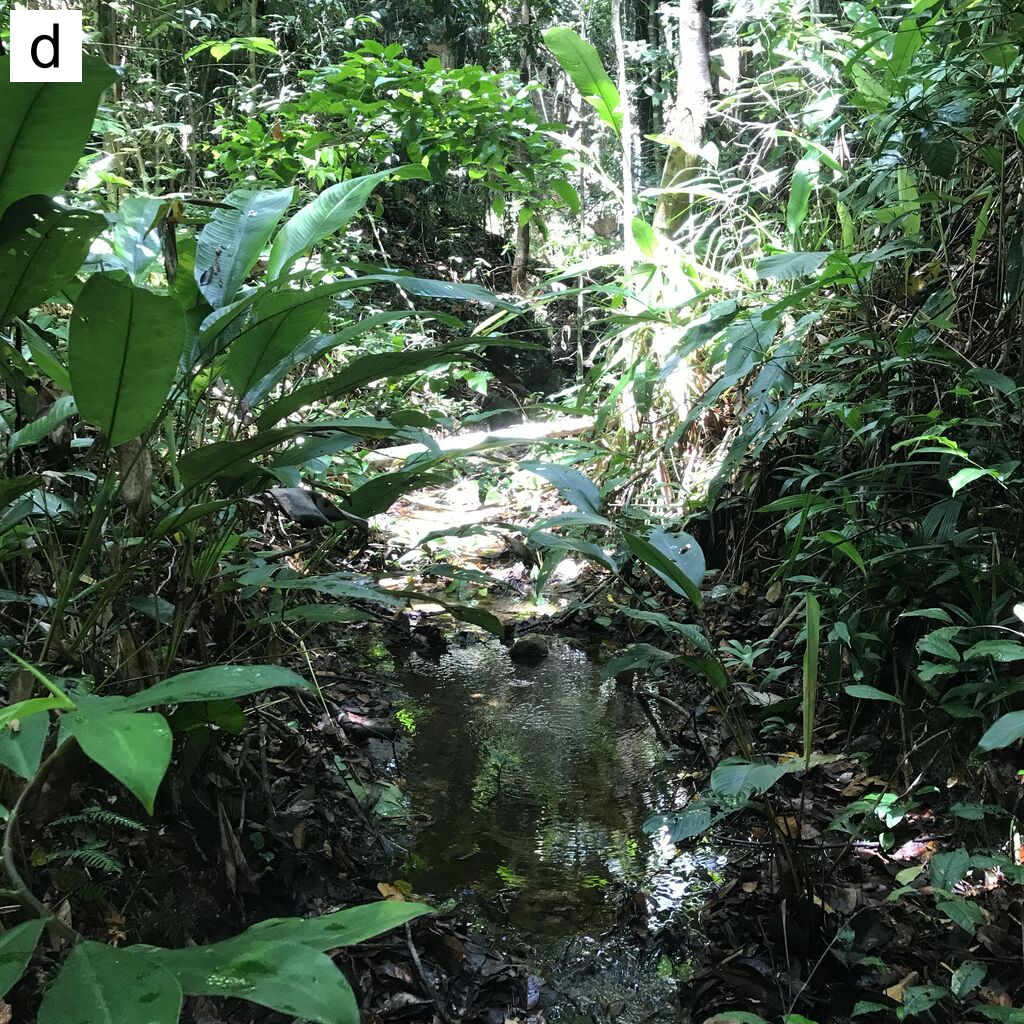
Seasonally flooded forest

**Figure 2e.**
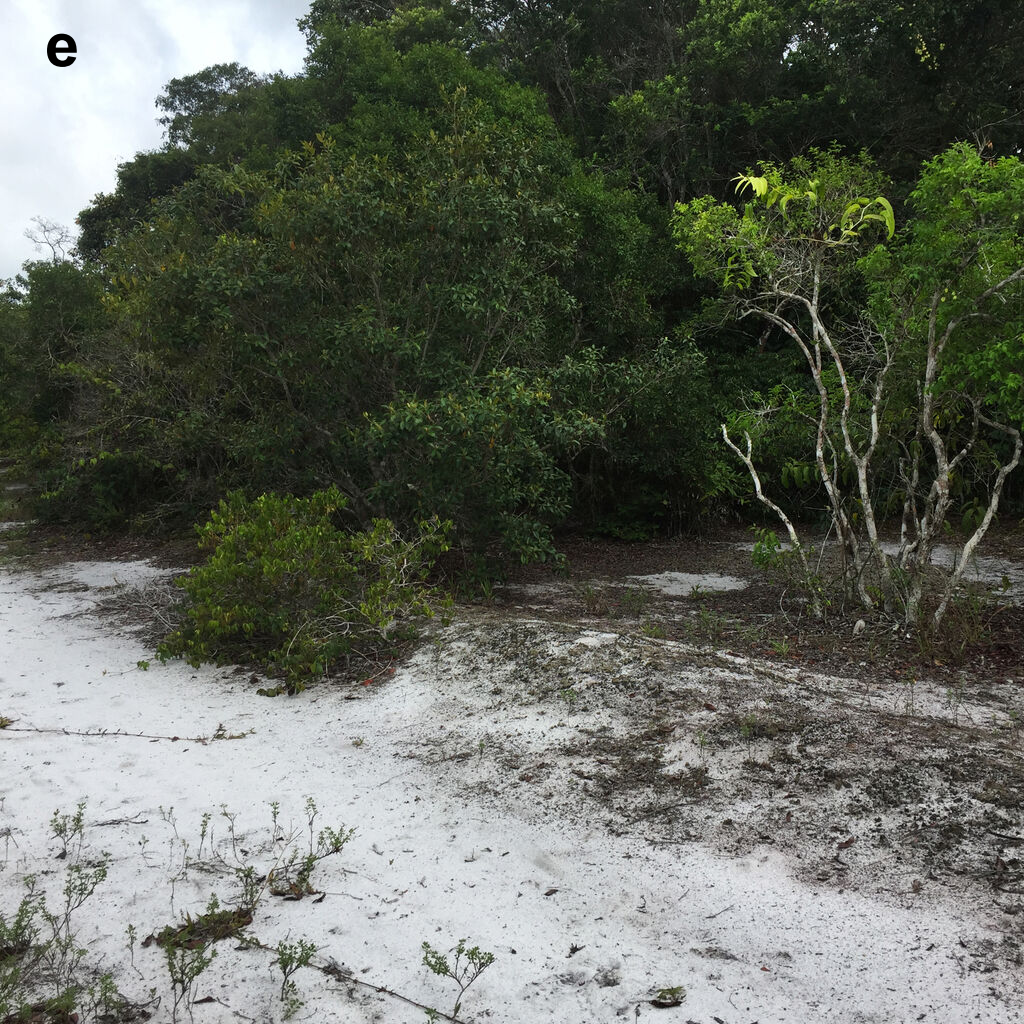
Mussununga

**Figure 2f.**
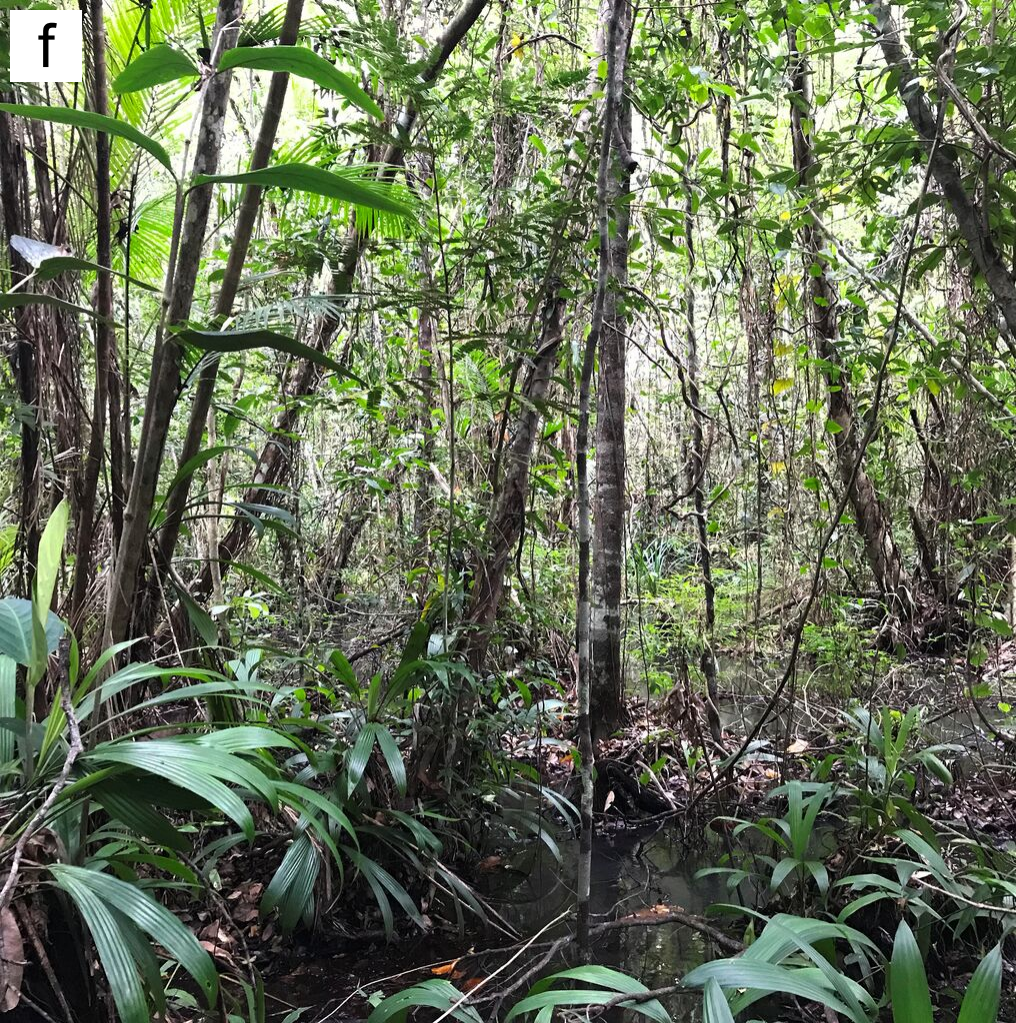
Flooding swampy area.

**Figure 5a.**
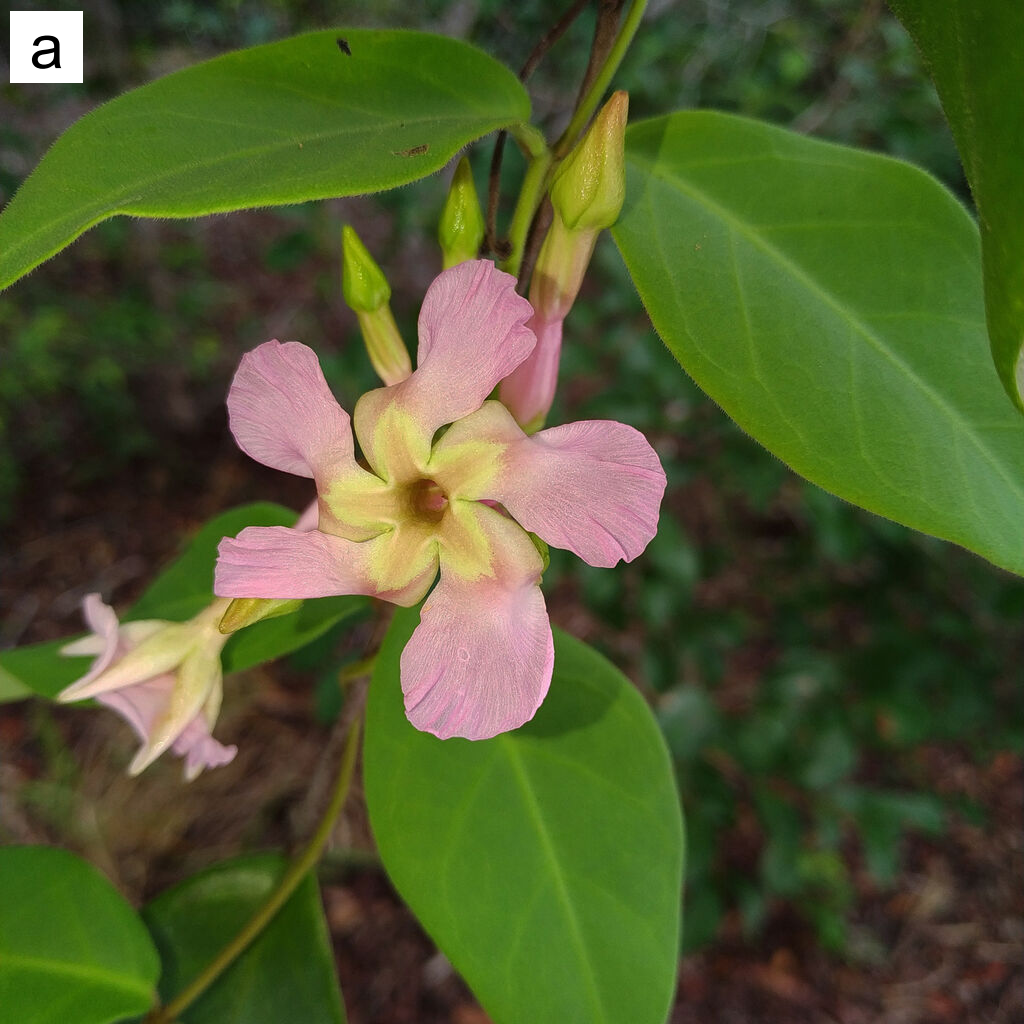
*Temnadeniaodorifera* (Apocynaceae)

**Figure 5b.**
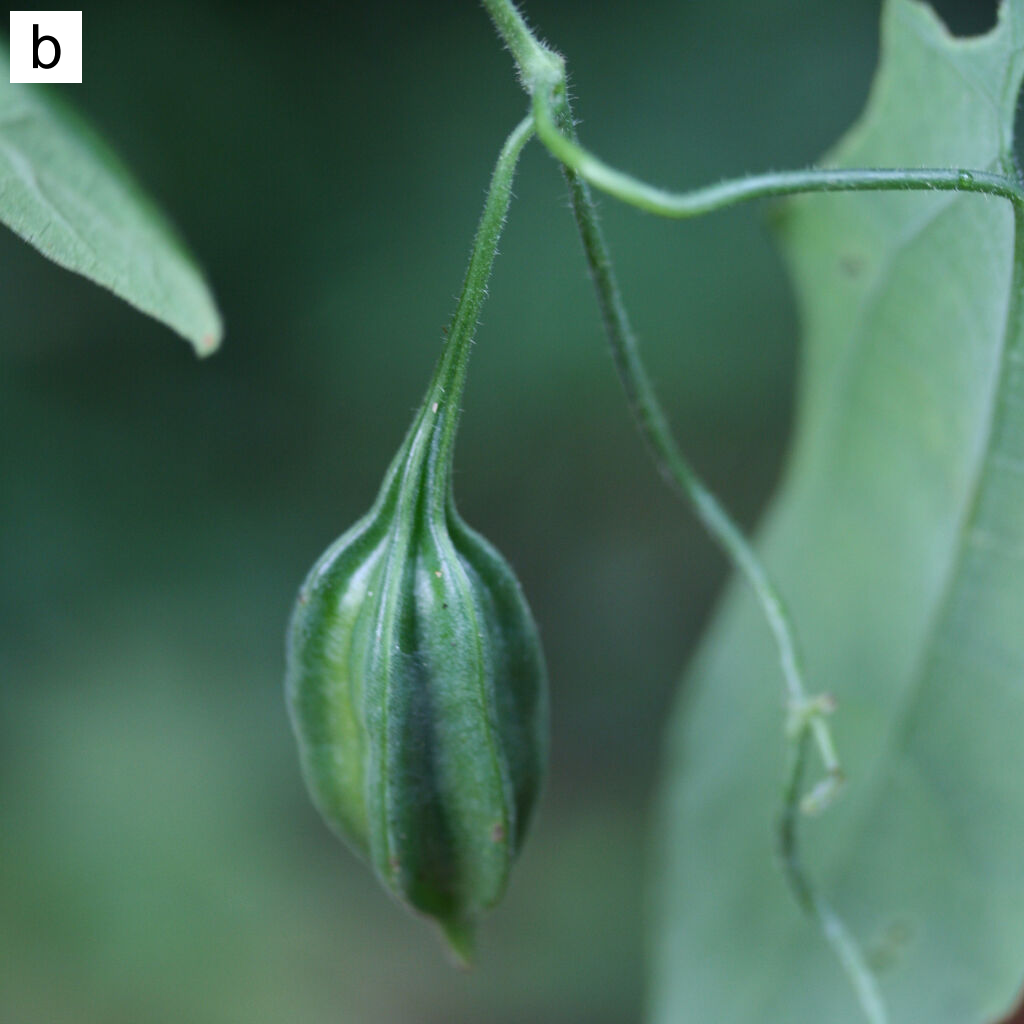
*Aristolochiapubescens* (Aristolochiaceae)

**Figure 5c.**
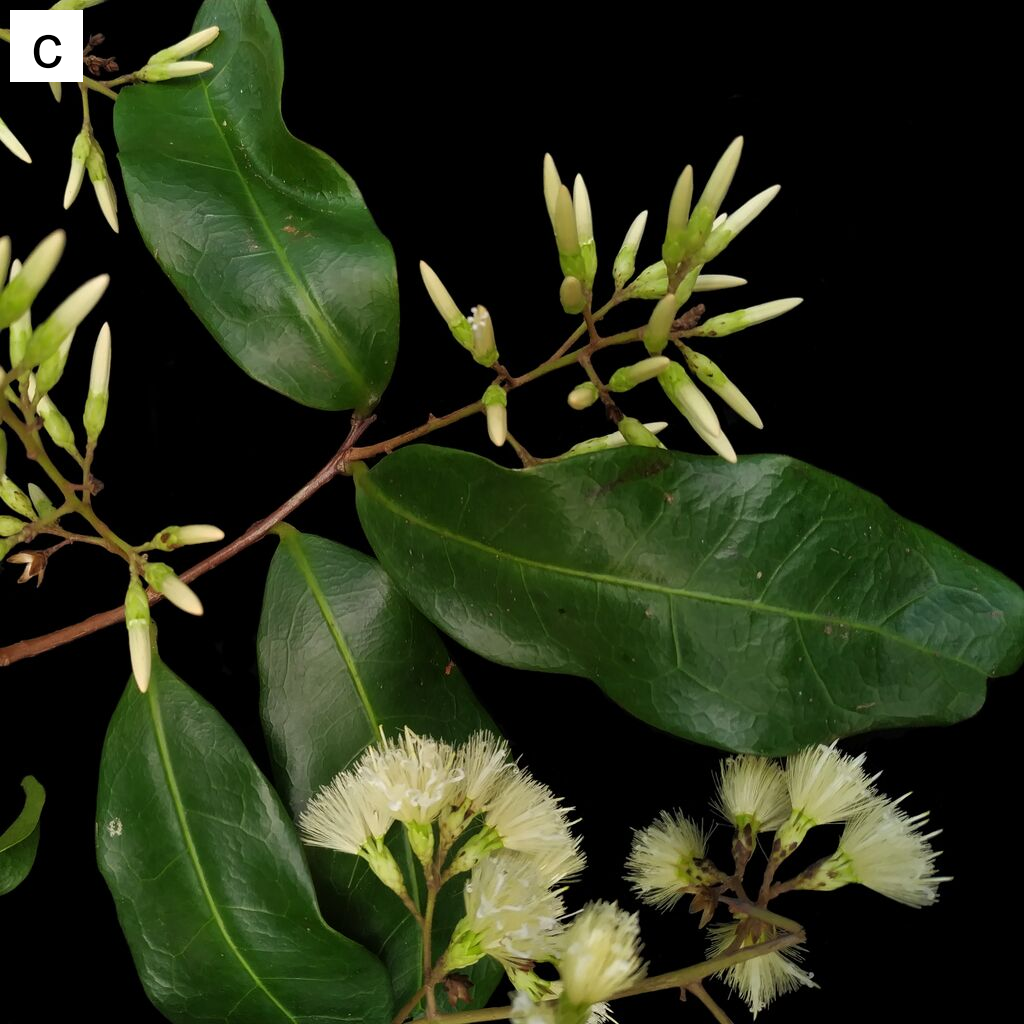
*Stifftiahatschbachii* (Asteraceae)

**Figure 5d.**
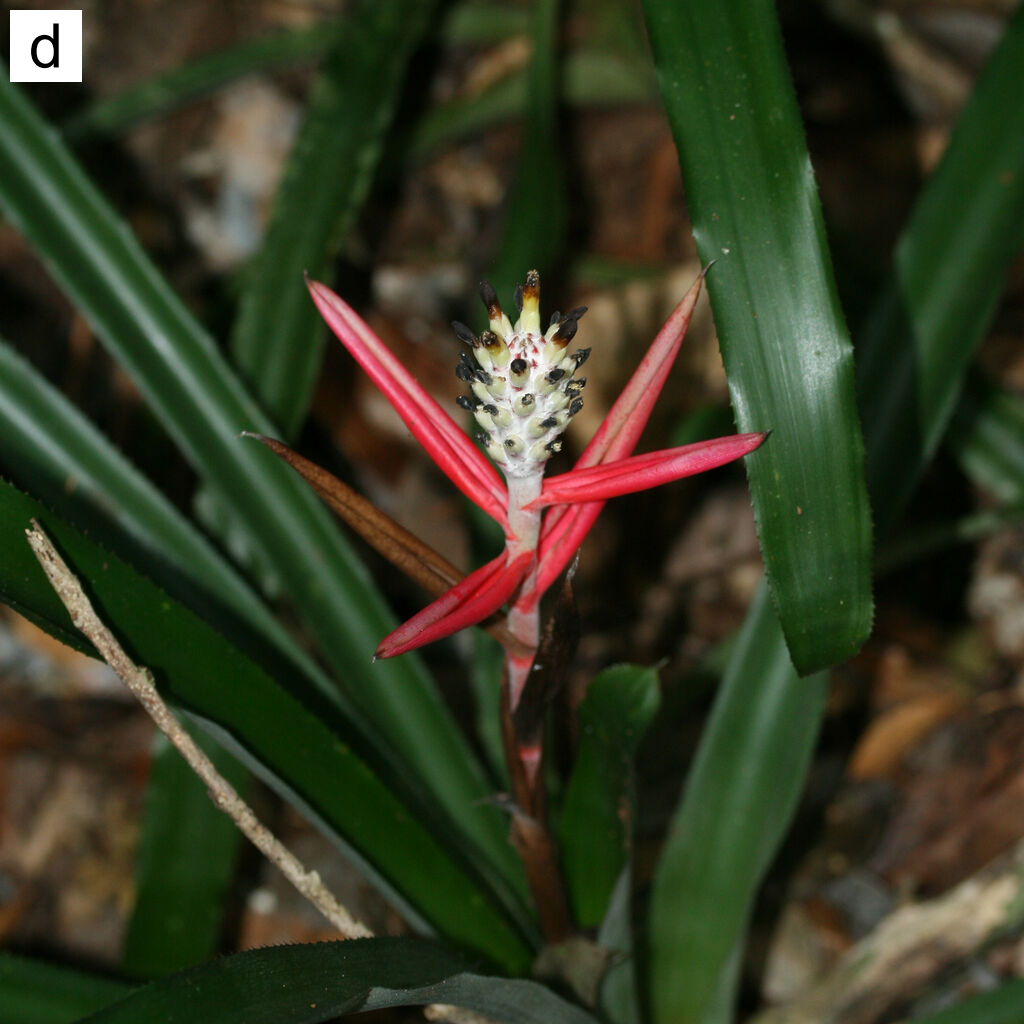
*Aechmeamaasii* (Bromeliaceae)

**Figure 5e.**
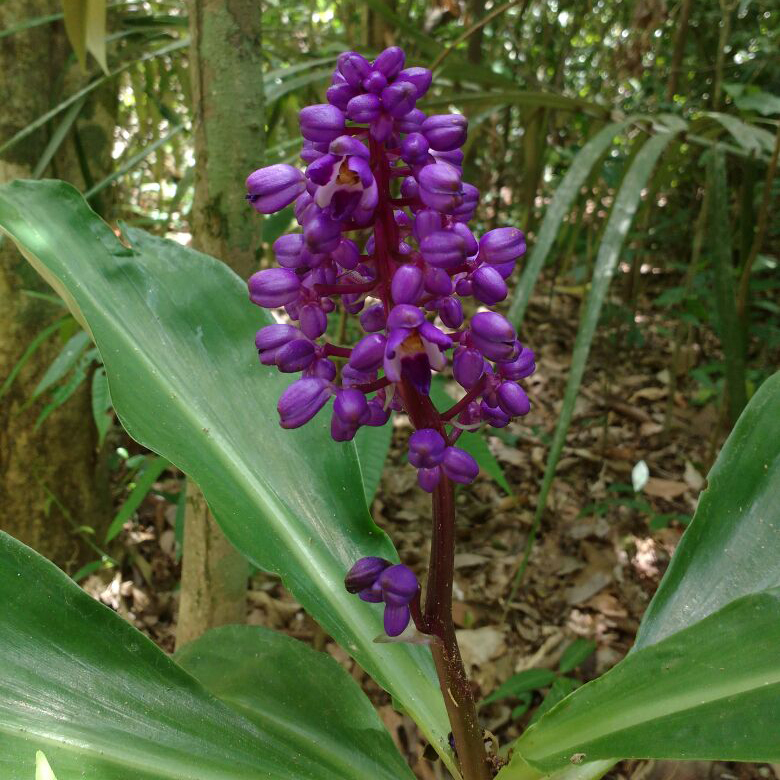
*Dichorisandraprocera* (Commelinaceae)

**Figure 5f.**
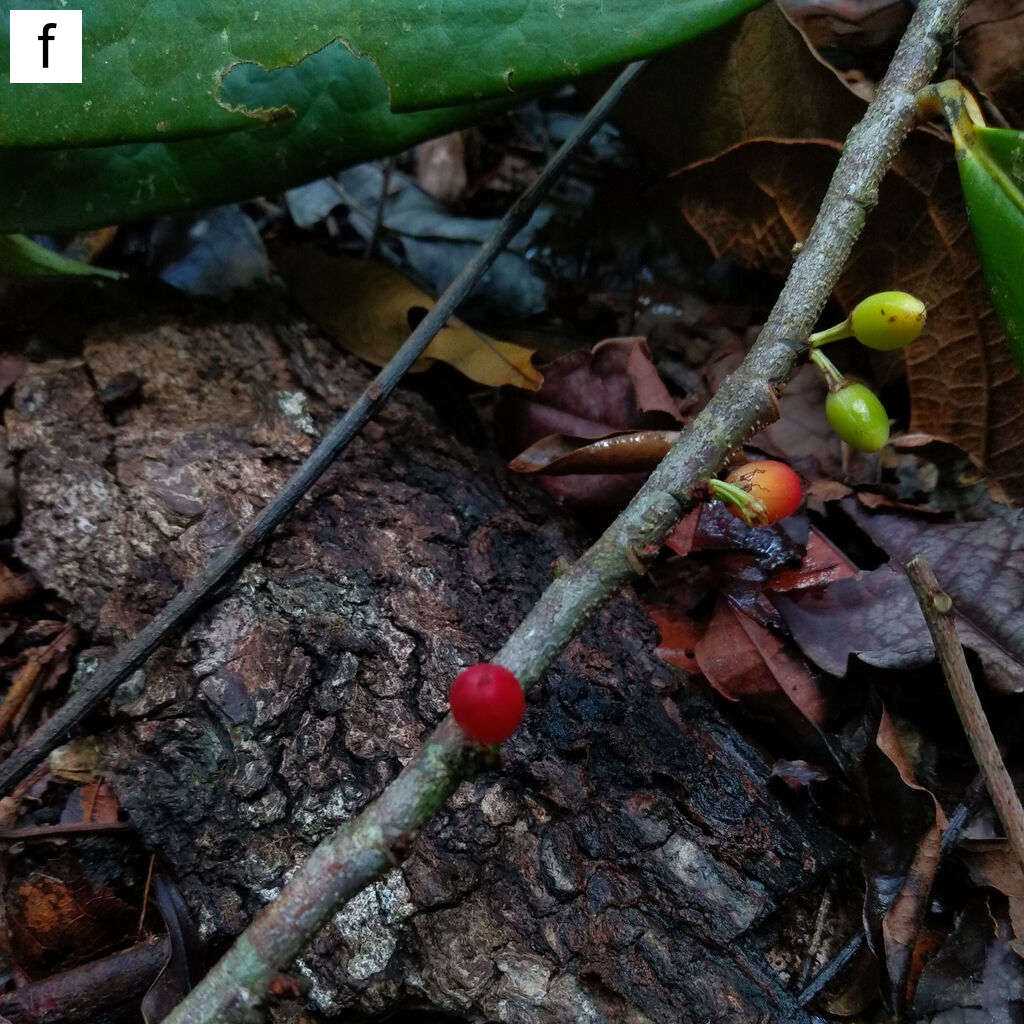
*Erythroxylumcolumbinum* (Erythroxylaceae).

**Figure 6a.**
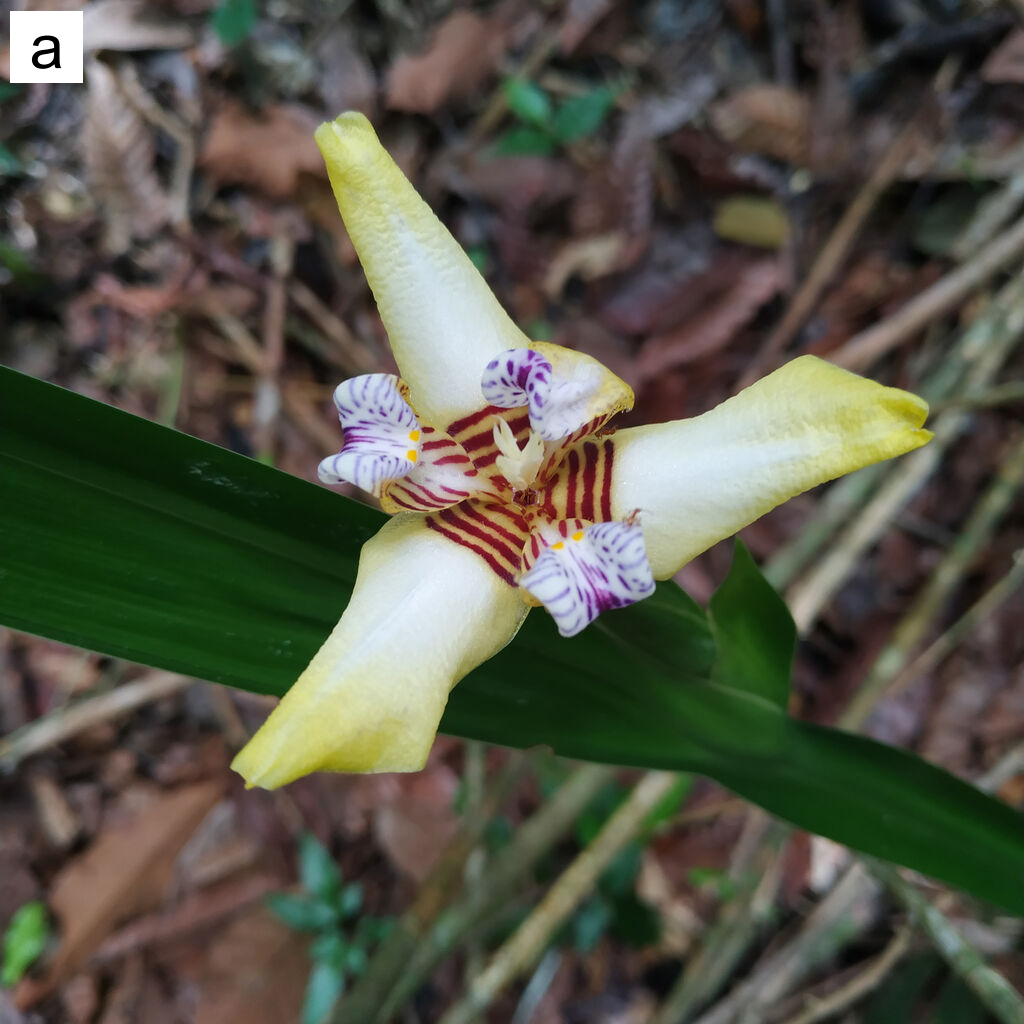
*Neomaricakollmannii* (Iridaceae)

**Figure 6b.**
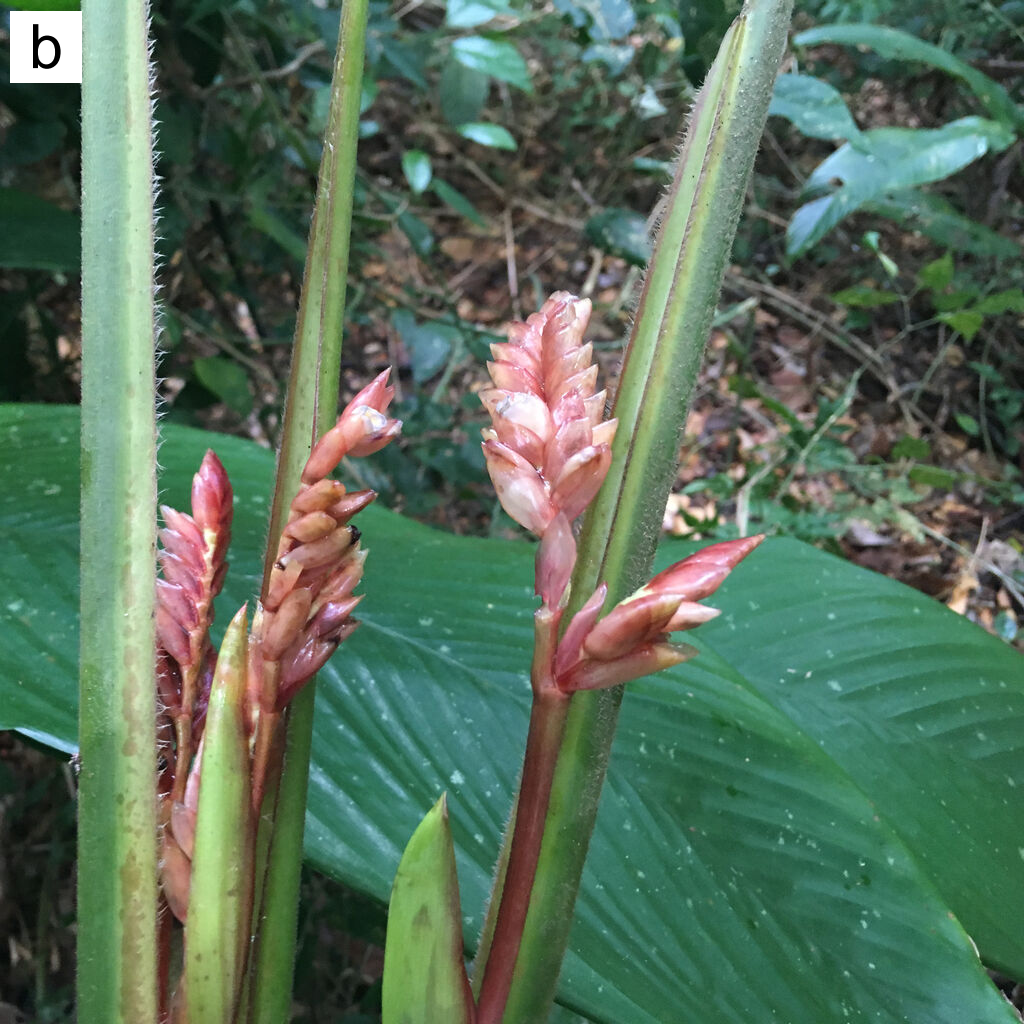
*Saranthecomposita* (Marantaceae)

**Figure 6c.**
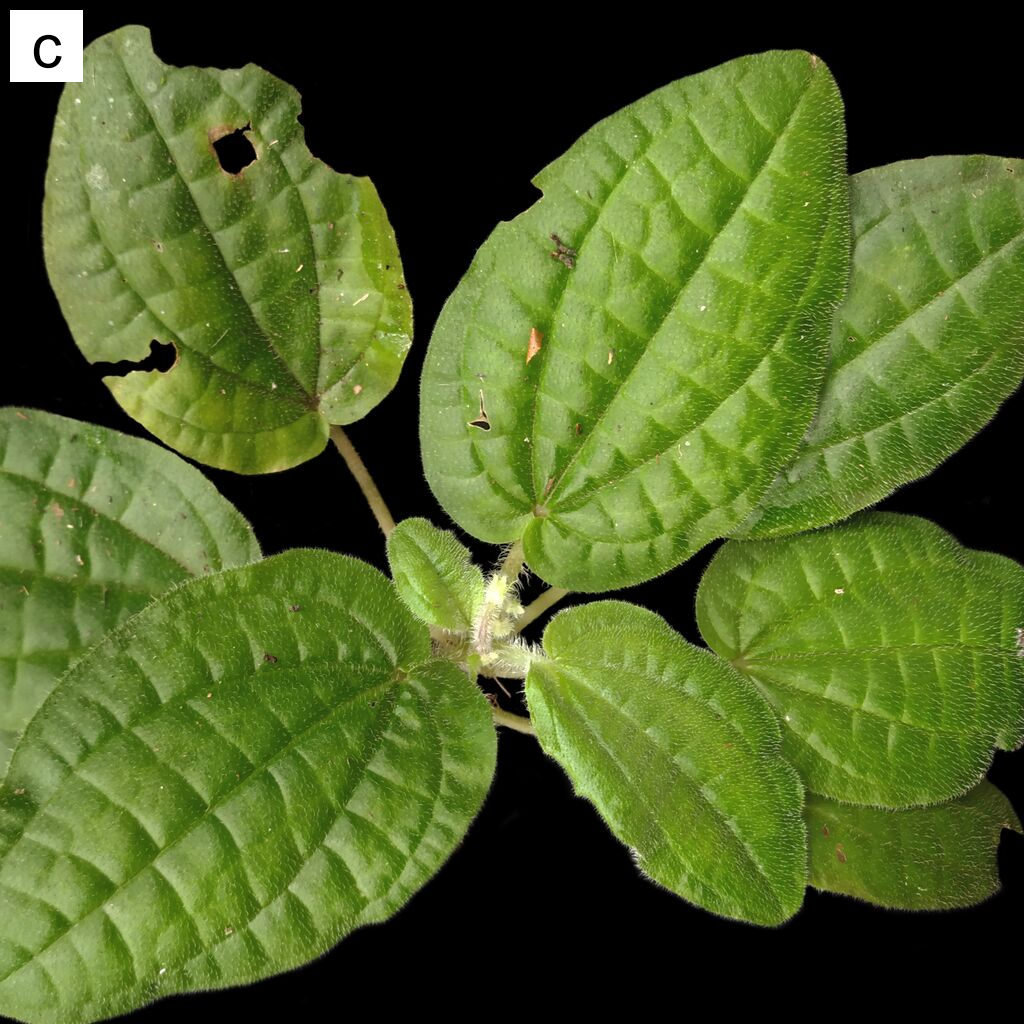
*Bertoloniamaculata* (Melastomataceae)

**Figure 6d.**
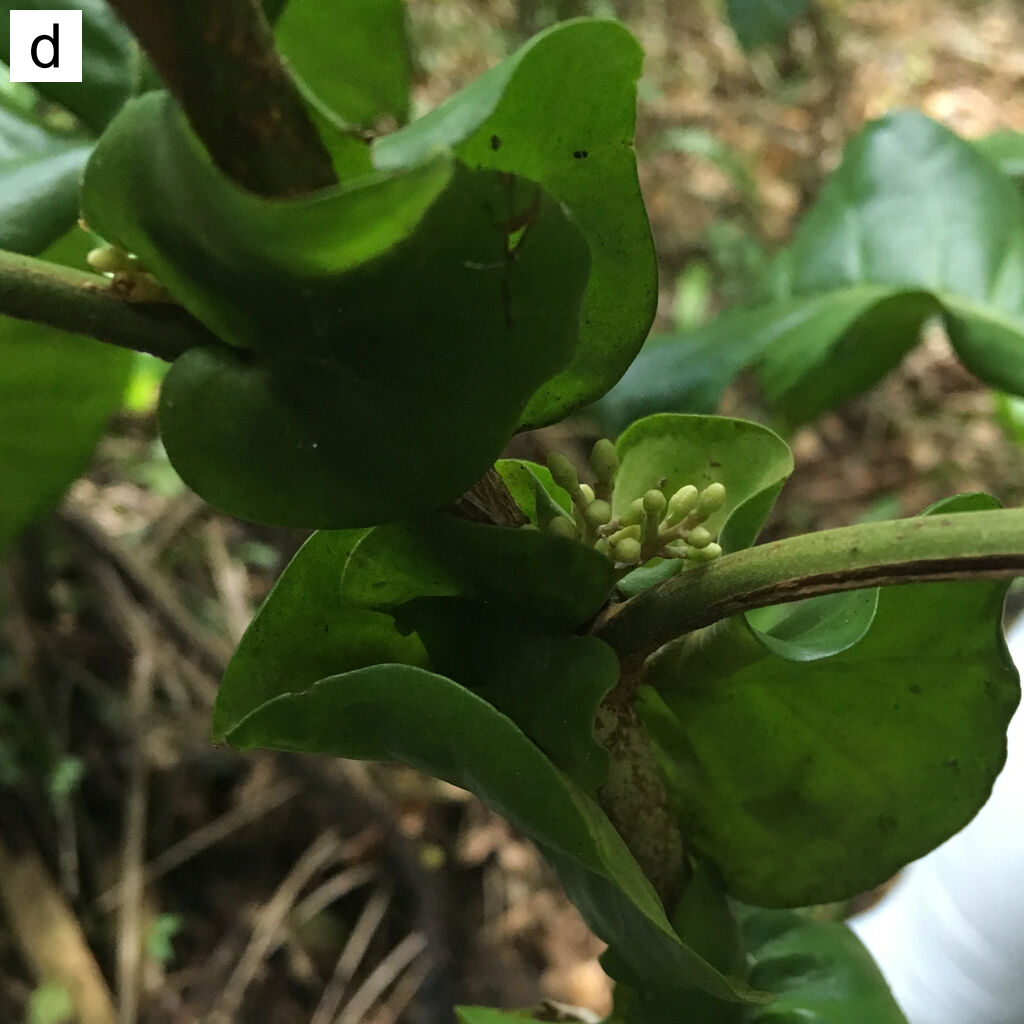
*Trichiliapseudostipularis* (Meliaceae)

**Figure 6e.**
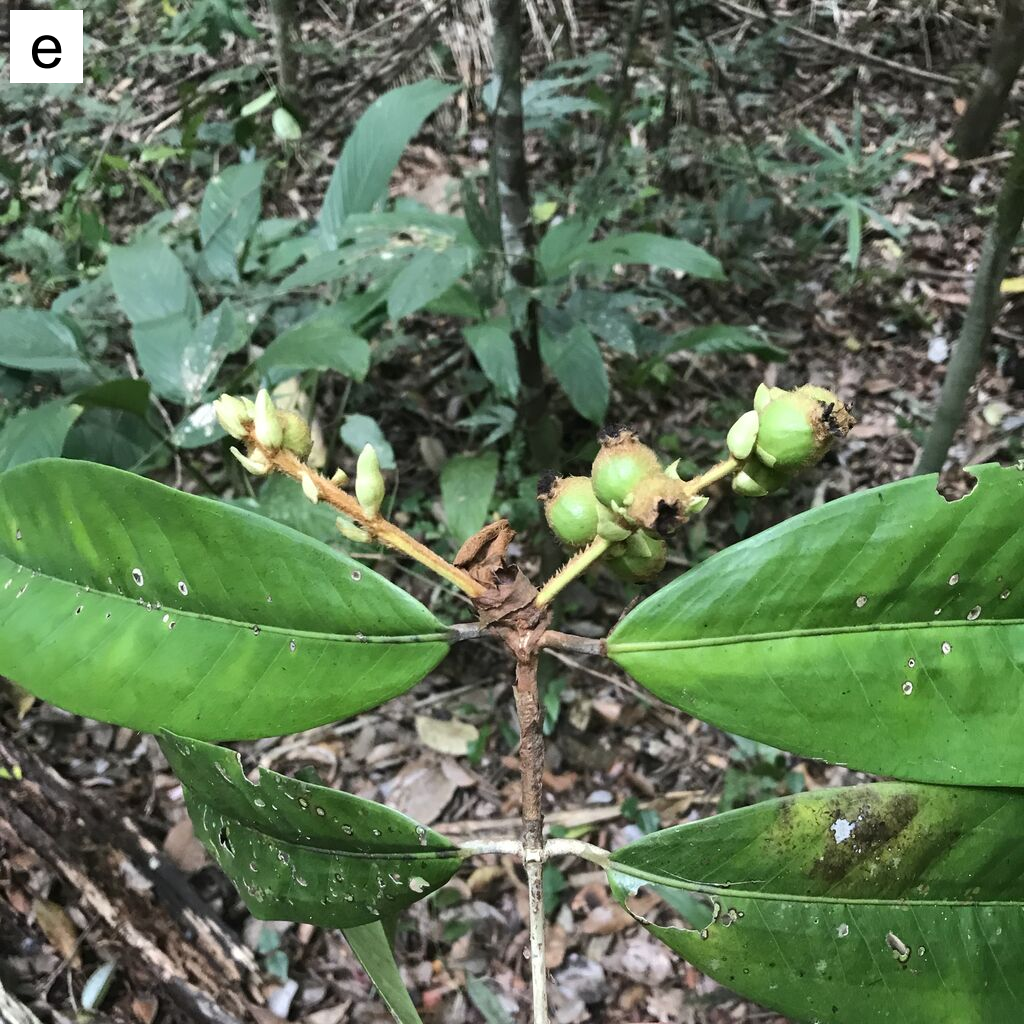
*Myrciasucrei* (Myrtaceae)

**Figure 6f.**
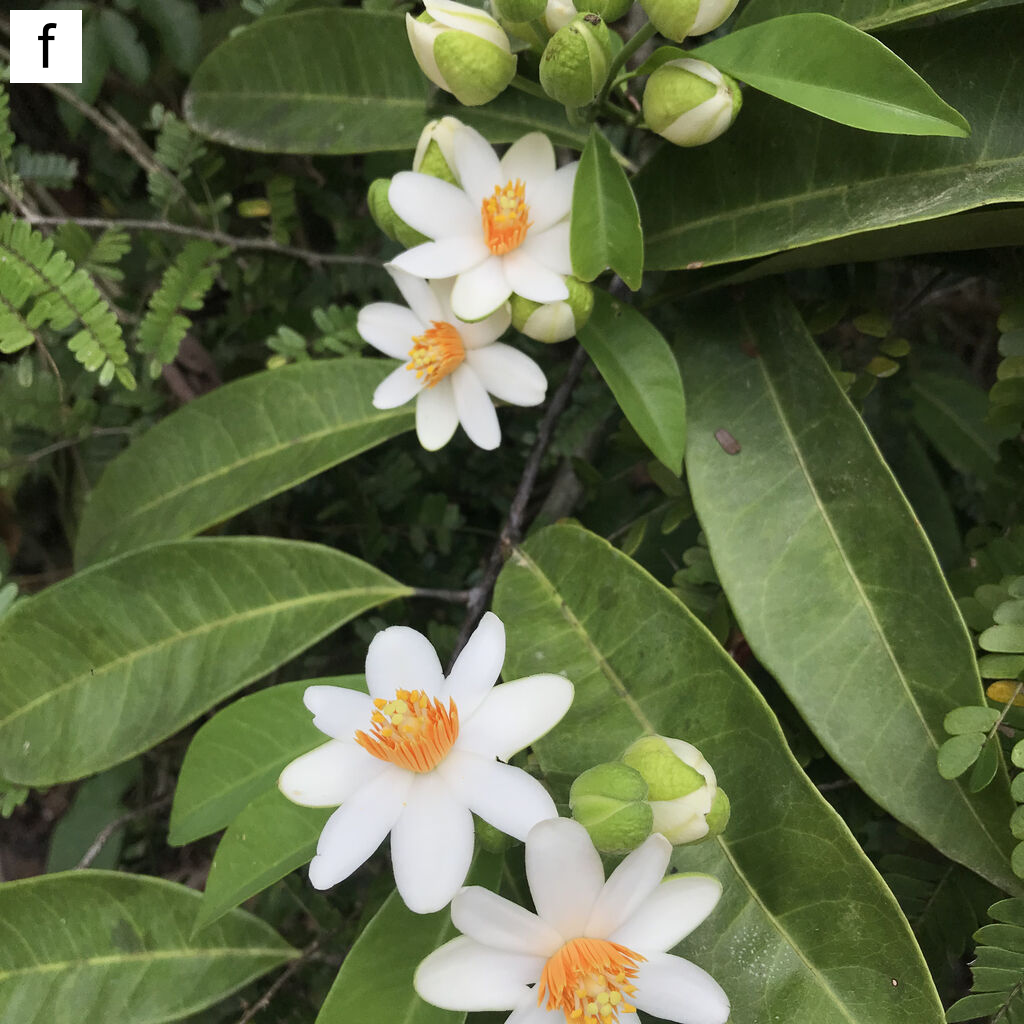
*Mitostemmaglaziovii* (Passifloraceae).

**Figure 7a.**
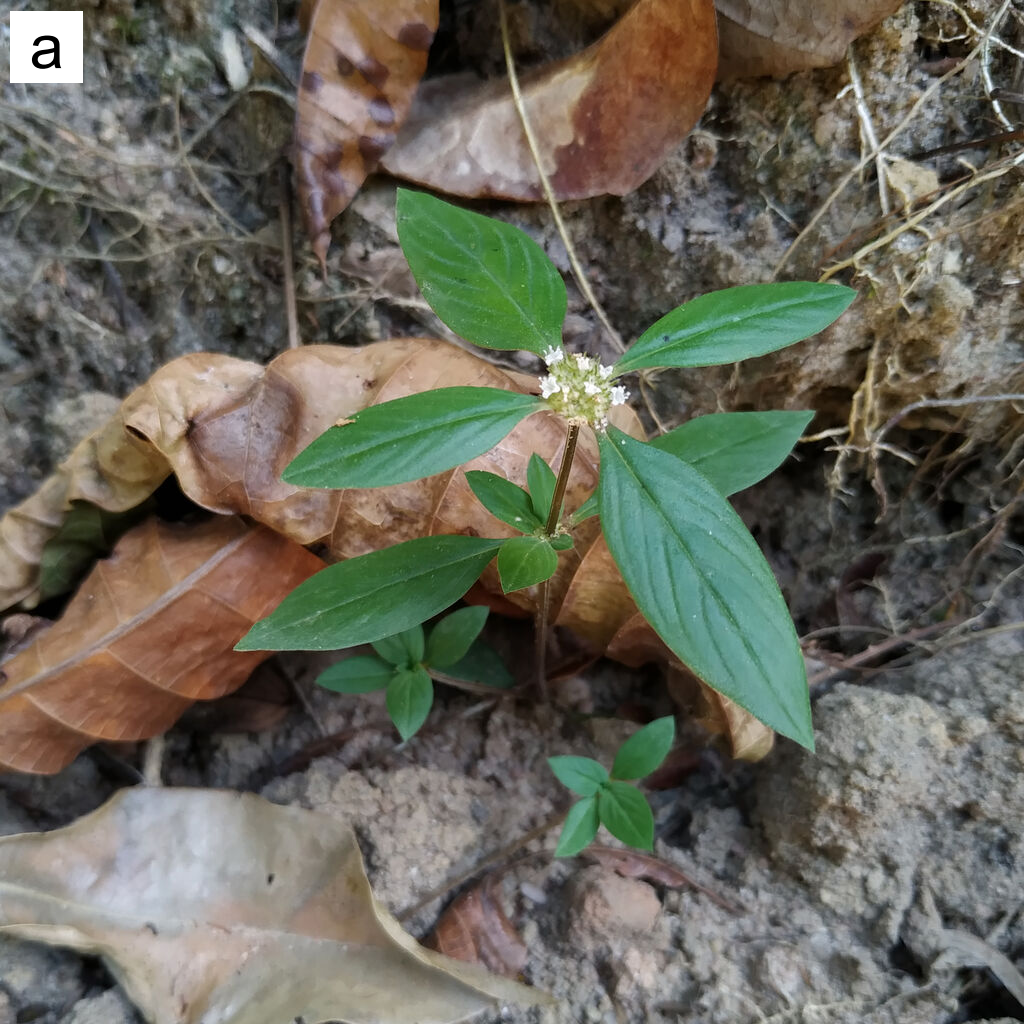
*Borreriacupularis* DC. (Rubiaceae)

**Figure 7b.**
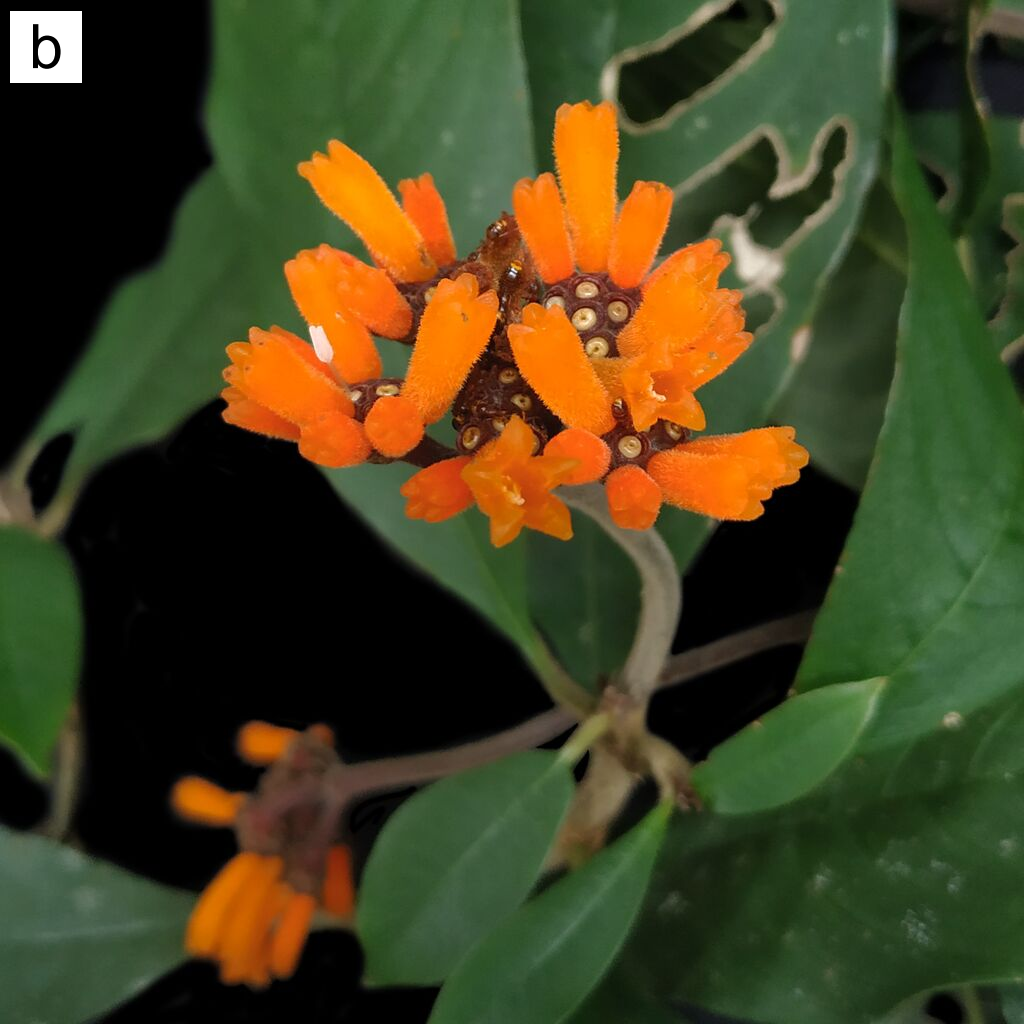
*Palicoureafulgens* (Müll.Arg.) Standl. (Rubiaceae)

**Figure 7c.**
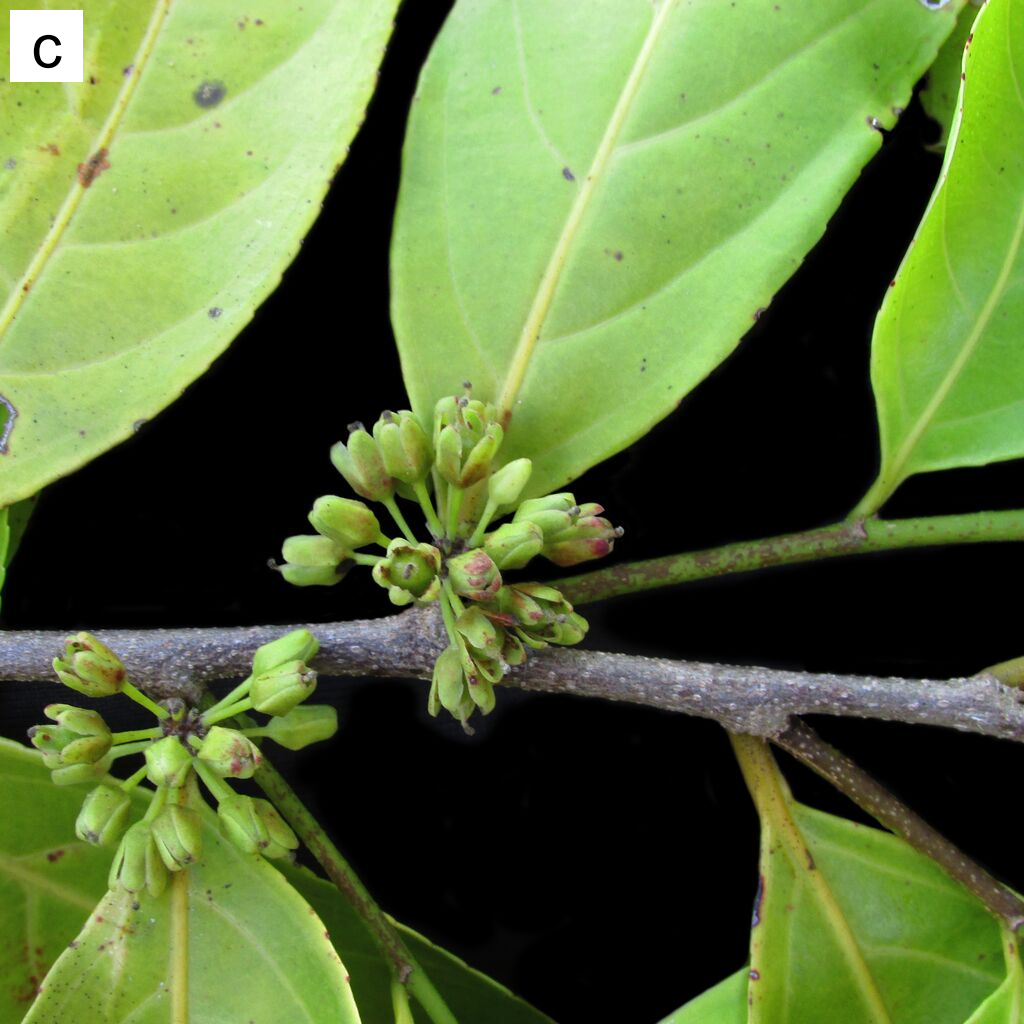
*Caseariaarborea* (Rich.) Urb. (Salicaceae)

**Figure 7d.**
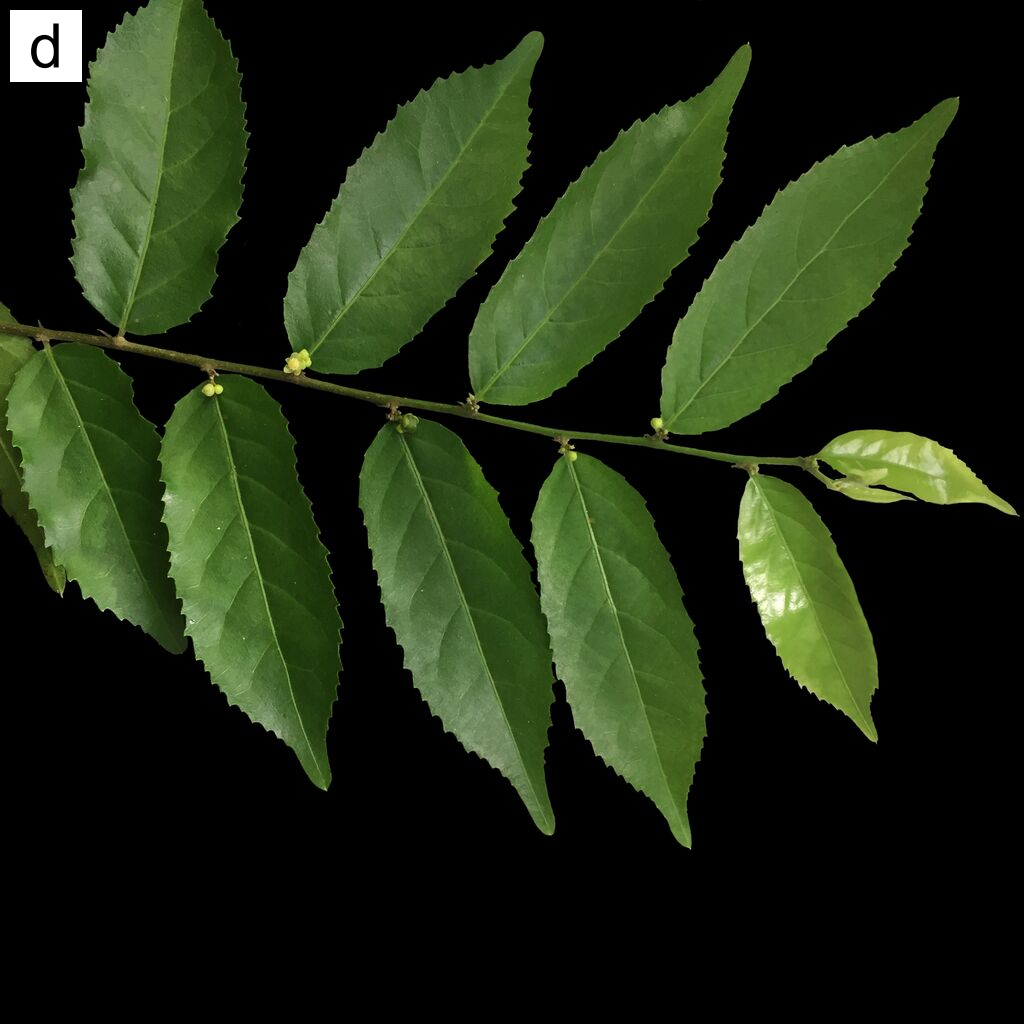
*Caseariasouzae* R. Marquete & Mansano (Salicaceae)

**Figure 7e.**
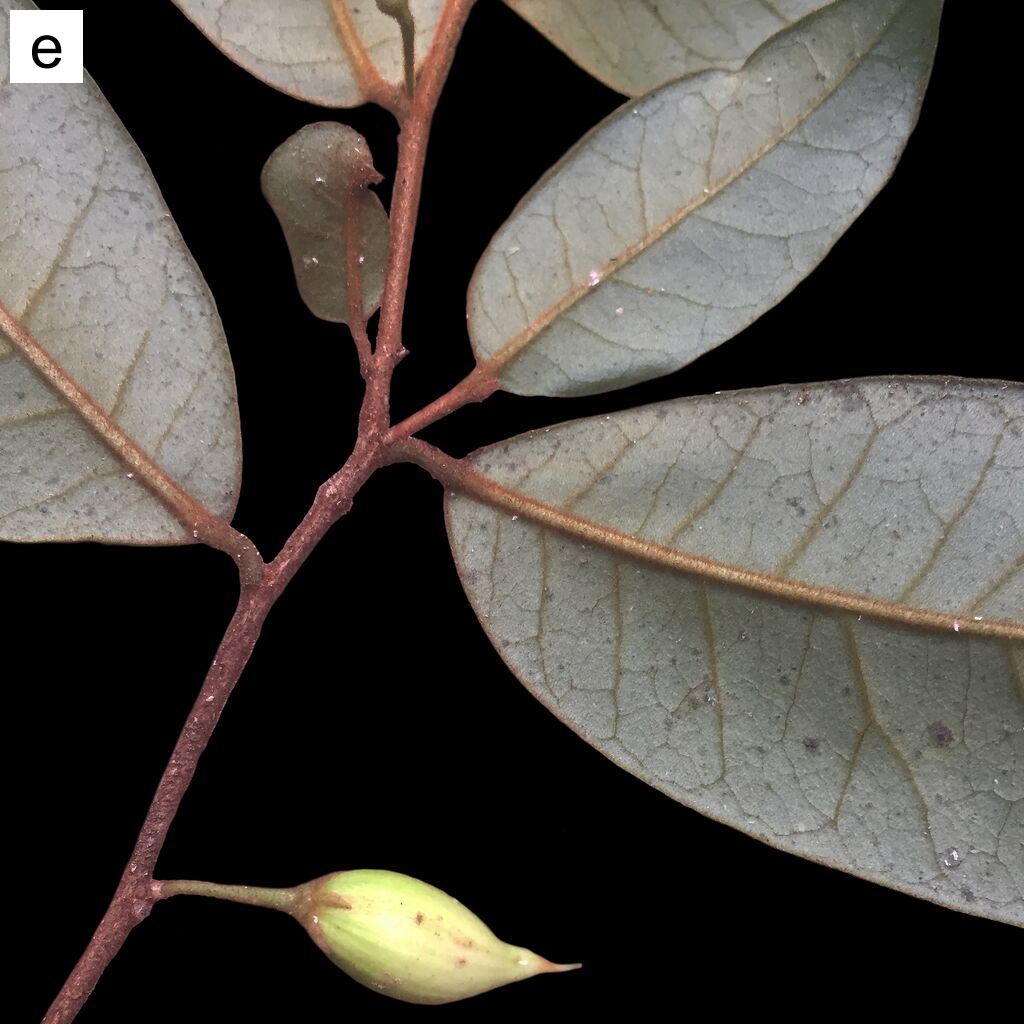
*Chrysophyllumlancisepalum* R.Lima (Sapotaceae)

**Figure 7f.**
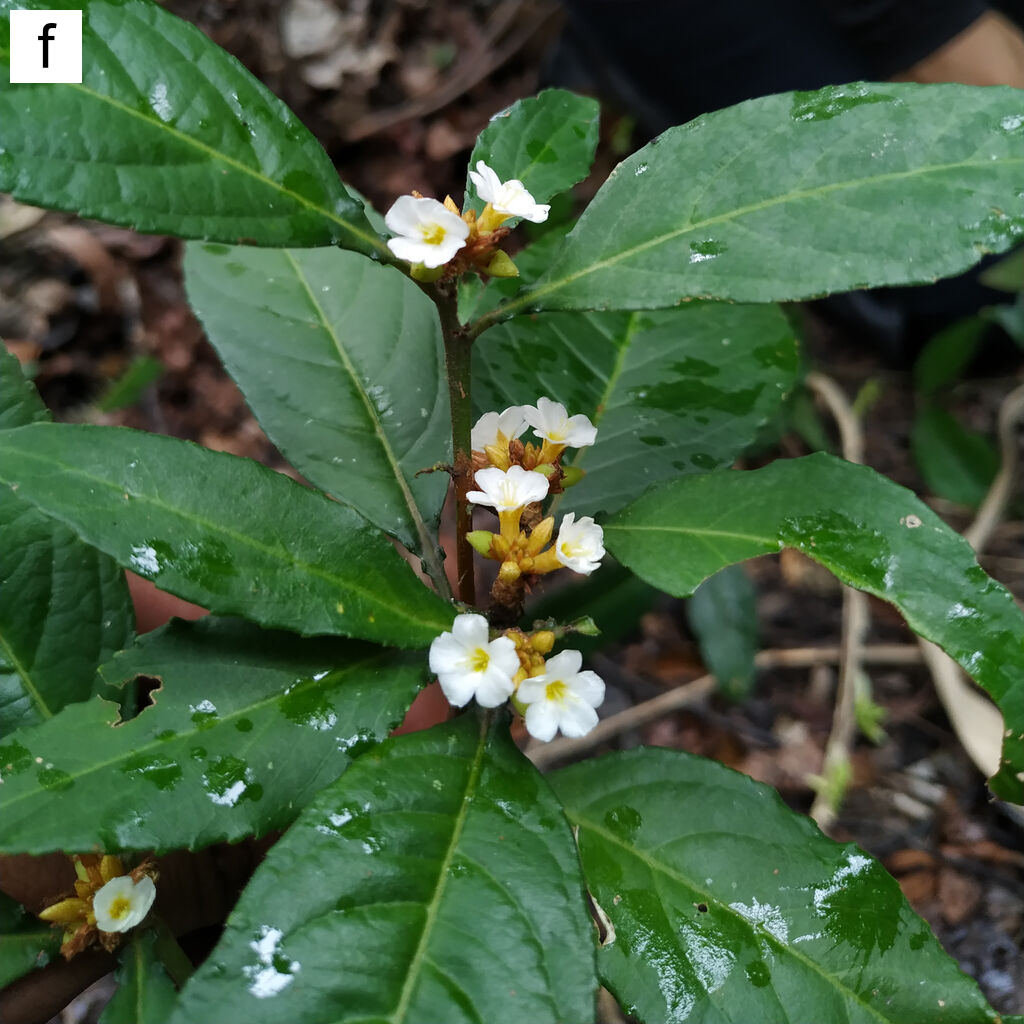
*Oxossiaspicata* (L.Rocha & Arbo) L.Rocha (Turneraceae).

**Table 1.** Threatened and Data Deficient species of plants occurring in "Flona do Rio Preto," Brazil with their respective groups, families and Red List category according to the Brazilian National Red List - CNCFLORA (CR = Critically Endangered, VU = Vulnerable, EN = Endangered and DD = Data Deficient).

Species	Red List category
Angiosperms	
Acanthaceae	
*Stenostephanuslobeliiformis* Nees	DD
Arecaceae	
*Euterpeedulis* Mart.	VU
Asteraceae	
*Piptocarpharobusta* G.M.Barroso	EN
Bignoniaceae	
*Handroanthusarianeae* (A.H.Gentry) S.O.Grose	EN
*Handroanthusriodocensis* (A.H.Gentry) S.O.Grose	EN
Burseraceae	
*Protiumheptaphyllum* (Aubl.) Marchand	DD
Chrysobalanaceae	
*Couepiabelemii* Prance	VU
*Couepiaschottii* Fritsch	EN
*Exellodendrongracile* (Kuhlm.) Prance	EN
Fabaceae	
*Apuleialeiocarpa* (Vogel) J.F.Macbr.	VU
*Ingaleptantha* Benth.	DD
*Melanoxylonbrauna* Schott	VU
Lauraceae	
*Williamodendroncinnamomeum* van der Werff	CR
Lecythidaceae	
*Carinianalegalis* (Mart.) Kuntze	EN
Marantaceae	
*Saranthecomposita* (Link) K.Schum.	VU
Meliaceae	
*Cedrelafissilis* Vell.	VU
Myristicaceae	
*Virolabicuhyba* (Schott ex Spreng.) Warb.	EN
Myrtaceae	
*Myrciaisaiana* G.M. Barroso & Peixoto	EN
Rubiaceae	
*Melanopsidiumnigrum* Colla	VU
*Palicoureafulgens* (Müll.Arg.) Standl.	VU
*Standleyakuhlmanni* Brade	EN
Sapotaceae	
*Manilkarabella* Monach.	EN
*Manilkaraelata* (Allemão ex Miq.) Monach.	DD
*Manilkaramultifida* T.D.Penn.	VU
*Pouteriabullata* (S.Moore) Baehni	EN
*Pouteriacoelomatica* Rizzini	VU
*Pouteriamacrocarpa* (Mart.) D.Dietr.	VU
*Pradosiaglaziovii* (Pierre) T.D.Penn.	DD
